# Freestanding 3D Multilayer Graphene Foams from Nanotextured Ni-Cu Templates

**DOI:** 10.3390/nano16150950

**Published:** 2026-08-01

**Authors:** Jaimon Chonedan Johnson, Nicolò Galvani, Piera Maccagnani, Alessandro Surpi, Nicola Gilli, Rita Rizzoli, Alessandro Gradone, Giulia Lorusso, Fabiola Liscio, Vittorio Morandi

**Affiliations:** 1 Dipartimento di Scienza Applicata e Tecnologia (DISAT), Politecnico di Torino, Corso Duca degli Abruzzi, 24, 10129 Torino, Italy; jaimon.chonedan@polito.it; 2 Istituto per lo Studio dei Materiali Nanostrutturati-Consiglio Nazionale delle Ricerche (ISMN-CNR Bologna), Via Piero Gobetti 101, 40129 Bologna, Italy; nicologalvani@cnr.it (N.G.); alessandro.surpi@cnr.it (A.S.); nicola.gilli@cnr.it (N.G.); fabiola.liscio@cnr.it (F.L.); 3 Istituto per lo Studio dei Materiali Nanostrutturati-Consiglio Nazionale delle Ricerche (ISMN-CNR), Strada Provinciale 35d, n. 9, 00010 Montelibretti, Italy; vittorio.morandi@cnr.it

**Keywords:** 3D graphene foam, multilayer graphene, chemical vapor deposition (CVD), porous metallic scaffolds, Ni-Cu alloy catalyst, hydrogen bubble templating, electrodeposition, template etching

## Abstract

Three-dimensional (3D) graphene foams are attractive as lightweight conductive scaffolds with large surface area and broadband light absorption but achieving reproducible porosity and preserving the architecture after metal-template removal remain challenging. Here we report a stepwise route to freestanding 3D multilayer graphene foams based on (i) hydrogen-bubble-assisted electrodeposition of porous Ni on Cu foils, (ii) time-controlled pre-annealing at 1000 °C to drive Cu diffusion and form porous Ni-Cu alloy templates, (iii) in situ graphene CVD at 1000 °C under fixed growth conditions, and (iv) wet etching to remove the metal scaffold without a polymer support. The influence of pre-annealing (0, 1, 3, and 7 h) on template evolution, graphene growth, and foam stability was systematically investigated via SEM, EDS, XRD and Raman studies. Before etching, Raman spectroscopy indicates low-defect graphenic coatings with locally heterogeneous few-layer-like to multilayer-like signatures. Only samples pre-annealed for at least 3 h preserved the porous 3D architecture after metal removal, indicating the formation of self-supporting graphenic networks with improved post-etch morphological stability. Raman and XRD analyses further revealed a progressive reduction in structural degradation, residual strain, and stacking disorder with increasing pre-annealing time. Among the investigated samples, the foams obtained after 3 and 7 h of template pre-annealing combined preserved 3D morphology with low sheet resistance (10–20 Ω/□), negligible optical transmittance (<5%), and strong broadband visible-light absorption (75–90%).

## 1. Introduction

Three-dimensional (3D) graphene-based foams have attracted considerable interest because they combine the chemical stability, electrical conductivity, and low density of sp^2^ carbon networks with an open, interconnected porous architecture. This structural arrangement provides large accessible surface area and efficient pathways for mass and charge transport, making graphene foams promising freestanding platforms for adsorption, catalysis, energy-related devices, sensing, and electromagnetic interference shielding [1,2,3,4,5,6,7,8,9,10].

Nevertheless, producing 3D graphene foams in a scalable and reproducible way remains challenging, especially when both the porosity of the starting template and the porous architecture integrity of the final carbon network must be preserved after metal removal [11,12,13].

This requirement makes the synthesis route particularly important. Among the available methods for graphene preparation, chemical vapor deposition (CVD) on transition-metal substrates remains one of the most effective approaches for obtaining high-quality graphene over extended areas. In CVD, the final carbon structure is determined by the combined effect of growth temperature, deposition time, hydrocarbon concentration, gas composition, pressure, cooling rate, and the interaction between carbon and catalytic metal [14,15]. The metal substrate plays a pivotal role as it catalyzes hydrocarbon decomposition and governs the dominant growth pathway through its carbon solubility, surface chemistry, and crystallographic structure. These aspects directly affect graphene coverage, uniformity, and number of layers [11,15,16,17].

The different behaviors of copper and nickel illustrate this point clearly. Copper has very low carbon solubility and typically promotes a surface-mediated growth process. Carbon species generated from the hydrocarbon precursor adsorbs and diffuse on the Cu surface, where they nucleate and expand into graphene domains. Once a continuous graphene layer covers the surface, further catalytic decomposition is largely suppressed, which makes Cu particularly suitable for monolayer graphene growth [18]. Nickel, by contrast, has a much higher carbon solubility at the temperatures typically used for CVD processes. Carbon can dissolve into the Ni bulk during growth and subsequently segregate or precipitate at the surface during cooling. This dissolution–segregation–precipitation mechanism often produces few-layer or multilayer graphene/graphitic coatings, with the final structure depending on carbon uptake, cooling rate, grain structure, and nucleation density [19].

Because Cu and Ni promote such different growth regimes, combining them into Ni-Cu alloy catalysts offers a useful way to tune graphene formation rather than simply choosing between monolayer-oriented and multilayer-oriented growth. By adjusting the Ni-Cu ratio, the carbon solubility, diffusion, segregation, and precipitation behavior of the catalyst can be modulated, thereby influencing graphene coverage, thickness, and layer number [20].

Huang and Ruoff proposed the use of Ni-Cu alloys as layer-tunable substrates for the growth of single-layer, bilayer, and multilayer graphene, showing how alloy composition can bridge the growth behavior of pure Cu and pure Ni [21]. In this view, the alloy is not passive support, but an active catalytic medium whose composition defines the balance between surface-mediated growth and bulk-mediated carbon precipitation.

This balance becomes even more delicate when the catalyst is shaped into a porous 3D scaffold rather than used as a flat substrate. In template-assisted CVD, the metallic scaffold defines the geometry that the graphene network will inherit, while the CVD process conformally coats the template surfaces with carbon. The final foam is therefore controlled not only by the graphene growth mechanism, but also by the pore size, interconnectivity, wall thickness, surface roughness, composition, and thermal stability of the metallic template. After graphene growth, the scaffold may be retained to form a hybrid metal/graphene structure, or it may be chemically removed to obtain a freestanding graphene network that replicates the original porous architecture. The latter case is particularly demanding because, once the metal is etched away, the graphene coating must be sufficiently continuous and connected to withstand template removal without collapsing or fragmentation [4,10,22].

A closely related approach was reported by Huang et al., who demonstrated the CVD growth of porous graphene foams in film form using Ni-Cu templates [23]. Their work showed that few-layer graphene walls can form low-density, highly porous, and electrically conductive networks with excellent performance in electromagnetic interference shielding and rapid organic-liquid adsorption.

Although previous studies have demonstrated the potential of Ni-Cu templates for producing porous graphene architectures, the role of the metallic template evolution prior to graphene growth remains insufficiently resolved. In particular, when porous Ni electrodeposited on Cu is subjected to high-temperature treatment, Cu diffusion, Ni-Cu alloy formation, microstructural relaxation, and changes in catalytic behavior occur simultaneously. Consequently, it remains unclear how the duration of this pre-annealing step governs the transition from an initially Ni-rich porous scaffold to an alloyed catalyst capable of producing a morphologically stable freestanding graphene network after metal removal.

**Figure 1:**
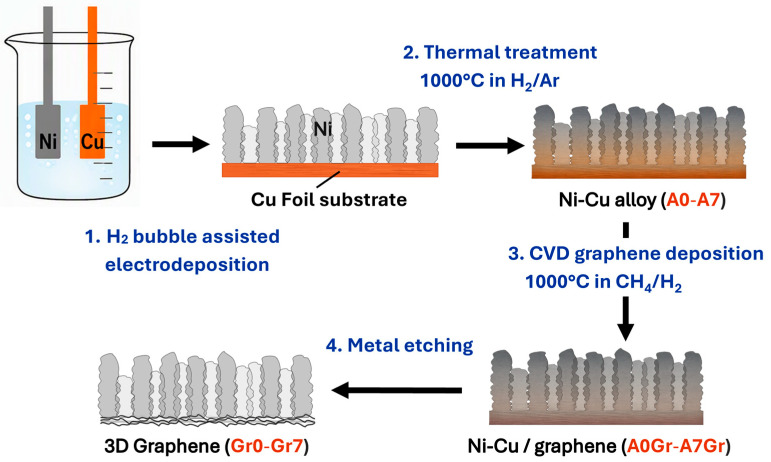
Schematic representation of the samples at the different synthesis stages: (1) porous Ni on Cu foil after electrodeposition, (2) Ni-Cu porous alloy after thermal treatment, (3) Ni-Cu porous alloy coated by CVD graphene, and (4) isolated 3D graphene after etching of the metal template.

Here, we address this issue through a stepwise strategy for fabricating freestanding 3D multilayer graphene foams from nanotextured Ni-Cu templates. Porous Ni films were first realized on Cu foils via hydrogen-bubble-assisted electrodeposition [24], and the pre-annealing duration was systematically varied to control Cu diffusion and Ni-Cu alloy formation. Graphene was then grown under identical CVD conditions for all samples, followed by wet removal of the metallic scaffold without polymer support. The overall fabrication sequence is schematically illustrated in Figure 1. By varying only the template pre-annealing time while keeping the graphene-growth parameters fixed, we directly correlate the structural and compositional evolution of the Ni-Cu scaffold with graphene organization and with the ability of the resulting carbon network to preserve its freestanding three-dimensional architecture after etching. The novelty of the work therefore lies in identifying pre-annealing-driven template alloying as the processing link between metallic-scaffold evolution, graphene growth, and post-etch foam stability.

The morphology, composition and structure of the samples were investigated across the different synthesis stages using SEM, EDS, and XRD, while Raman spectroscopy is used to evaluate disorder, defect/edge-activated contributions, and the multilayer character of the graphene network. Finally, electrical and optical properties were carried out to evaluate conductivity and light-harvesting potential.

## 2. Materials and Methods

### 2.1. Nickel Electrodeposition on Copper Foil

Copper foils (Cu-OFHC, 99.99% purity, 0.025 mm thickness; Goodfellow, Huntingdon, United Kingdom) were employed as the working substrate, while nickel foils (99.99% purity, 0.05 mm thickness, Goodfellow) were used as the counter electrode. The Cu foils were mechanically polished with rough and fine sandpaper to remove oxide layers. Both Cu and Ni foils were cut into 2 × 3 cm samples and cleaned in an ultrasonic bath using, sequentially, acetone, isopropanol, and DI water, to remove organic contaminants and surface residues.

Electrodeposition was carried out using a VA156 potentiostat (Neutron Laboratory, Bologna, Italy). The Cu foil was connected as the cathode (−ve) and the Ni foil as the anode (+ve), with the electrodes kept 2 cm apart and immersed vertically in an electrolyte solution containing 0.2 M NiCl_2_ and 2 M NH_4_Cl and deionized water (DI, resistivity > 18 MΩ·cm) with approximately 2 × 2 cm^2^ exposed area. Following deposition, the samples were thoroughly rinsed with DI to remove residual electrolyte and dried at room temperature.

### 2.2. Time-Controlled Ni-Cu Alloying

As-deposited porous Ni-on-Cu samples were used as a metal-template control and were not exposed to high temperature. For alloying, samples were placed in a hot-wall CVD furnace and dried overnight under vacuum at room temperature to remove moisture. Pre-annealing was performed at 1000 °C under a reducing H_2_/Ar atmosphere while varying only the pre-annealing dwell time (*t*_anneal). The investigated times were *t*_anneal = 0 h (**A0**), 1 h (**A1**), 3 h (**A3**), and 7 h (**A7**). Each condition was reproduced in at least three independent samples (*n* ≥ 3).

### 2.3. In Situ Graphene Growth Under Fixed CVD Conditions

Graphene deposition was carried out immediately after pre-annealing, using the CVD setup at atmospheric pressure and at 1000 °C. A CH_4_/H_2_ mixture (10:30 sccm) was introduced for 5 min (**A0** sample), and the growth time was defined from the onset of CH_4_ flow at the setpoint temperature. No intentional stabilization step was used; the gas-switching transient lasted only a few seconds and corresponded to valve switching (opening CH4 and closing Ar). After growth, the furnace was cooled overnight to room temperature under a slightly reductant H_2_/Ar flow (10:150 sccm) to prevent oxidation. For the graphene-growth series (**A0**–**A7**), the only variable is *t*_anneal (0, 1, 3, 7 h), while all growth parameters were kept constant.

### 2.4. Template Removal via Wet Etching

After morphological and structural characterizations, the graphene-coated Ni-Cu samples were subjected to chemical etching to isolate freestanding 3D graphene. The samples were first immersed in a pre-heated FeCl_3_ solution (40% FeCl_3_ diluted 1:3 in deionized water) at 80 °C for 2 h. Then, to eliminate residual impurities, the samples were soaked for 30 min in a 1:3 mixture of hydrochloric acid (37% HCl) and deionized water solution, pre-heated to 80 °C. Finally, the samples were thoroughly rinsed with deionized water and dried overnight on glass slides under ambient conditions in a fume hood.

### 2.5. Characterization

Scanning electron microscopy (SEM) high-resolution imaging was performed using a field-emission SEM LEO 1530 and a field-emission SEM-FIB CrossBeam 350 (Carl Zeiss GmbH, Oberkochen, Germany). Energy-dispersive X-ray spectroscopy (EDS), integrated with the SEMs systems, was employed to determine the elemental composition and distribution within the porous structures.

X-ray diffraction (XRD) measurements were carried out using a SmartLab 9 kW (Rigaku Corporation, Tokyo, Japan) diffractometer equipped with a rotating Cu anode. Specular θ/2θ and Grazing-Incidence (GI-XRD) geometries were used to collect complementary bulk- and surface-sensitive diffraction information from the porous Ni-Cu/graphene system. In the 3D porous scaffolds, GI-XRD enhances the relative contribution from the outer regions of the network; however, the complex morphology prevents a direct conversion of the incidence angle into an absolute value of the probing depth. Standard scans were acquired in a 1D configuration using a HyPix-3000 detector without receiving Soller slits, providing an angular resolution of approximately 0.02° in specular geometry. High-resolution XRD (HRXRD) scans were collected in a 0D configuration using a two-bounce Ge(400) monochromator, reaching an angular resolution of approximately 0.007° in specular geometry. HRXRD was restricted to specular geometry because of the large beam footprint and limited sample flatness under GI conditions.

Raman spectroscopy measurements were conducted at room temperature using a InVia Qontor Microscope (Renishaw, Wotton-under-Edge, United Kingdom) equipped with a 532 nm (Green) Laser (1800 gr/mm grating) to extract information on the structural quality, defect-related contributions, and local graphenic thickness and stacking regime before and after removal of the metallic scaffold. The thickness assessment was based on the combined analysis of the ration between the 2D and G band intensities (*I*_2D_/*I*_G_) and the shape, position, asymmetry, and FWHM of the 2D band. Because of the curvature and structural heterogeneity of the three-dimensional network, these parameters were used as qualitative or semi-quantitative indicators and were not converted into a direct measurement of the average number of graphene layers.

To obtain a first assessment of the graphene coverage, Raman measurements were initially performed using a 20× objective, providing a spatial resolution of ~2 μm, comparable to the characteristic dimensions of the scaffold structures. Five random areas were selected on the outer surfaces, pore regions were intentionally excluded, as focusing on these areas is unreliable due to the spatial resolution being comparable to the pore size.

After etching the Ni-Cu scaffold, Raman maps were executed on different samples of 3D graphene. A 50× long working distance objective lens was employed to focus laser excitation on the sample. Raman mapping and spectra were performed over 10 × 10 μm^2^ regions corresponding to a full focused crest of the 3D structure with a step size of 1 μm, resulting in a total of 121 probed points, with spatial resolution of ~1 μm. Samples are 1 × 2 cm^2^ and the mapped area was selected within the central region to avoid edges and ensure spatially uniform and statistically reliable spectral analysis. The laser power density on the sample was maintained lower than 1 mW/μm^2^ to avoid laser-induced heating and damage.

The acquired spectra were processed using a home-developed python script based on LMFIT library [25]. The baseline was fitted with a second-order polynomial [26] using anchor points obtained from local minima selected within 30 cm^−1^ wide subsections of the spectral regions away from the peaks. Full description of the multi-peak fitting procedure is reported in the Supplementary Information.

Electrical measurements were carried out at room temperature and ambient atmosphere using a Karl Süss probe station equipped with four Keithley 238 (KeithIey Instruments, Inc., Cleveland, OH, USA) source monitor units (SMU) connected to the probe through the switching matrix Keithley 707 (with 7072 and 7174 semiconductor cards). The graphene samples were characterized by measuring their surface sheet resistance obtained using the four-point probe method. The probe spacing (*s*) was fixed at 500 μm for all measurements. The graphene samples had rectangular dimensions of 1 × 2 cm^2^, and thickness of 25 microns. Under these conditions, the sample thickness (*t*) was much smaller than the probe spacing (*t* ≪ *s*), while the lateral dimensions (*d*) were significantly larger than *s* (*d* ≫ *s*), so that the sheet resistance can be expressed using the following formula [27]: *Rs* = *ρ*/*t* = π/ln2 (Δ*V*/*I*) = 4.53 (Δ*V*/*I*).

Optical properties were characterized using a broadband UV–Vis–NIR fiber-optic system, which includes an DH-2000 dual lamp (deuterium and halogen) (Ocean Optics, Dunedin, FL, USA) as the light source and an fiber-optic spectrometer (Avantes, Apeldoorn, The Netherlands) as the detector. An external integrating sphere setup in a vertical top–bottom configuration was used to collect diffuse reflectance and transmission, ensuring accuracy for low-density, high-surface-area samples.

## 3. Results

### 3.1. Electrodeposition Mapping and Selection of an Optimal Porous Ni Scaffold

A series of porous nickel-on-copper samples—hereafter referred as Ni-Cu—were realized via electrodeposition as described in Section 2.1. Depositions were carried out applying currents *I* of 1.0 A (0.25 A/cm^2^), 1.6 A (0.40 A/cm^2^), and 2.4 A (0.6 A/cm^2^), with deposition time *t* ranging from 30 s to 7 min. The electrodeposition process affects both sides of the samples: the front side, directly facing the Ni foil electrode, and the back side. SEM imaging in Figure S1 (Supporting Information) reveals for the front side a reproducible morphological evolution with increasing deposition severity: (i) nucleation of Ni clusters, (ii) dendritic growth, and (iii) a fully developed 3D porous scaffold shaped by concurrent hydrogen evolution. As expected, the back side (Figure S2) exhibits a highly discontinuous growth. Specifically, near the edges—where the electrical current is still effectively delivered—a relatively thick coating, as on the front side, is observed. Instead, in the central region of the back side, nucleation is very limited, resulting in a sparse and poorly developed structure. Hereafter, unless otherwise specified, we will refer exclusively to the Ni-Cu scaffold formed on the front side of the samples.

**Figure 2:**
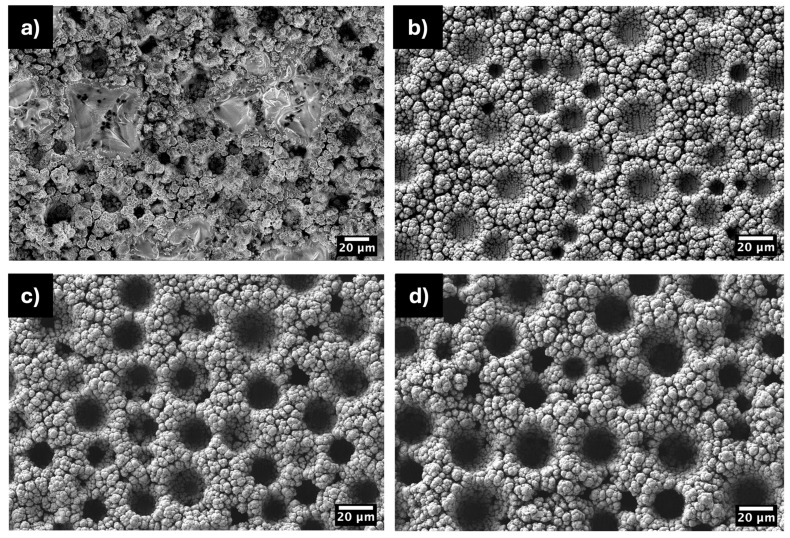
SEM image of a Ni-Cu scaffold obtained via three minutes of Ni electrodeposition at different currents: (**a**) *I* = 1.0 A; (**b**) *I* = 1.0 A, 1.6 A; (**c**) *I* = 2 A (**d**) *I* = 2.4 A.

Based on pore distribution quality, reproducibility, and film homogeneity, the optimal deposition time was identified as *t* = 3 min (Figure S1), while a current of *I* = 2.0 A (Figure 2) was selected as the optimal scaffold for subsequent alloying and graphene growth.

**Figure 3:**
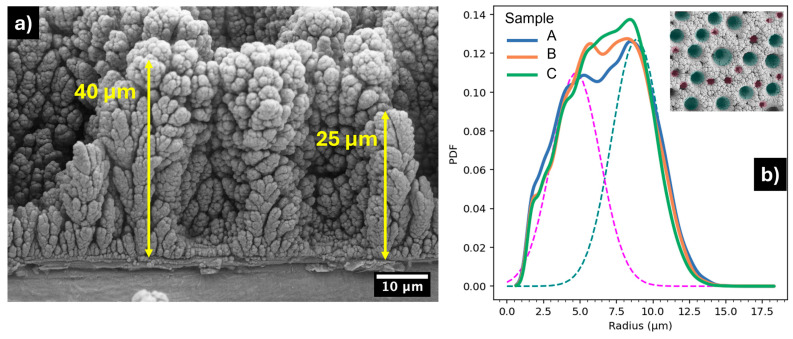
(**a**) Cross-sectional SEM image of a Ni-Cu scaffold fabricated at an electrodeposition current of *I* = 2 A (current density of 0.50 A/cm^2^) and *t* = 3 min (**b**) pore size distributions obtained for three different batches of the sample, showing highly reproducible bimodal profiles. Dashed lines indicate Gaussian fits. (Inset) Top-view SEM image of a representative portion of sample A, highlighting the coexistence of large (green) and small (purple) pores.

To evaluate the pore size distribution of the Ni-Cu scaffolds, a systematic workflow, described in detail in the Supplementary Information, was applied to SEM micrographs corresponding to 2 A (Figure 3) and 2.4 A (Figure S3) samples. Porosity was quantified as the ratio of void area to the total cross-sectional area, providing a reliable estimate of the open volume fraction within the scaffold. For three batches of the sample deposited at 2 A for 3 min, namely: A, B and C, the analysis, reported in Figure 3, revealed a highly reproducible porous structure across different experiments. The pore size distribution displayed a clear bi-disperse nature, which could be modeled as the superposition of two Gaussian distributions with mean radii *μ* = 8.88 and 4.72, standard deviations *σ* = 1.69, and 1.67, and with relative weights of 0.54 and 0.46, respectively. This indicates the coexistence of two characteristic pore populations within the Ni-Cu scaffold.

### 3.2. Time-Controlled Alloying (A0–A7): Cu Diffusion and Structural Evolution

With these parameters fixed, we prepared a series of Ni-Cu alloys by thermally pre-annealing the samples at 1000 °C in a reducing H_2_/Ar atmosphere. The pre-annealing time was varied to 0, 1, 3, and 7 h (**A0**, **A1**, **A3**, **A7**) to investigate the progressive Ni-Cu interdiffusion, from the Ni-rich scaffold to a nearly homogenized alloy. The selected durations capture both the early stages of alloying and the approach to compositional stabilization.

**Figure 4:**
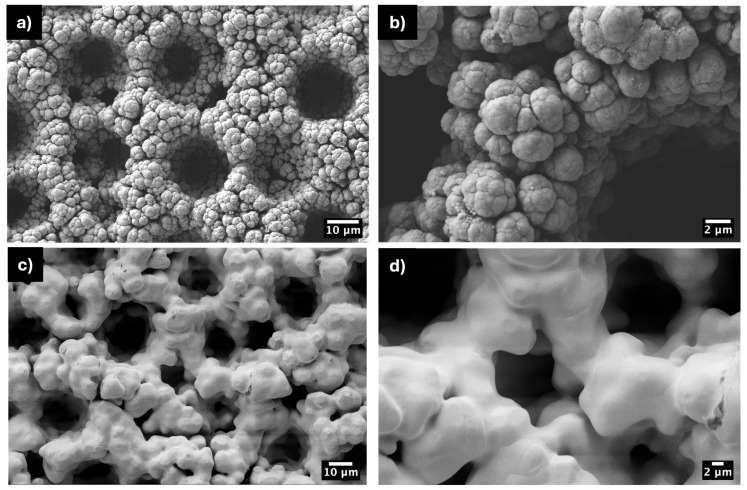
SEM images of a Ni-Cu scaffold fabricated with *I* = 2 A (0.50 A/cm^2^) and *t* = 3 min before (**a**,**b**) and after (**c**,**d**) 3 h pre-annealing.

SEM images show that the high-temperature treatment for **A3** sample “melts” or smoothens the morphology of the dendrites that compose the nanostructures, even though the overall macroporous scaffold remains remarkably intact and uncollapsed (Figure 4b–d). The same result is obtained for other samples (Figure S5).

**Figure 5:**
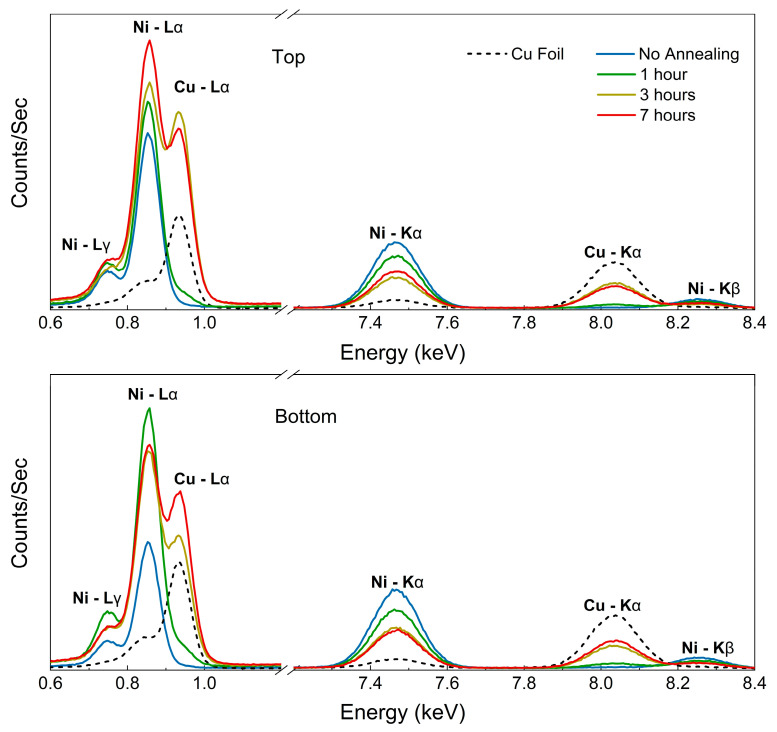
Energy-dispersive X-ray spectroscopy (EDS) spectra as a function of the pre-annealing duration at (**Top**) and (**Bottom**) of the porous Cu-Ni alloys. For comparison, the spectrum collected from the copper foil substrate is also included.

The compositional evolution was investigated using EDS measurements collected at different vertical positions of the porous scaffold, namely near the surface of the scaffold (Top) and just above the interface between the electrodeposited Ni and the Cu foil (Bottom). In the as-deposited Ni-Cu scaffold, only the Cu L signal originating from the underlying Cu foil was detected, while the Cu K signal was absent, confirming that Cu was not present within the electrodeposited Ni layer and that the porous scaffold was initially Ni-rich. Upon pre-annealing, the Cu K signal progressively appeared and increased in intensity, indicating Cu diffusion from the substrate into the Ni network. After 1 h, the Cu content increased to approximately 10 wt%, while after 3 h it reached values in the 40–45 wt% range. Further annealing up to 7 h produced only minor variations, with Cu contents remaining within the 30–45 wt% range depending on the probed region. These qualitative evaluations are related to the K peaks to reduce the experimental error related to the superposition of the L peaks. Cross-sectional EDS maps confirmed this trend and showed a residual vertical compositional gradient, with higher Cu content near the bottom of the scaffold, as expected from the Cu foil acting as the diffusion source (Figure 5 and Figure S6).

**Figure 6:**
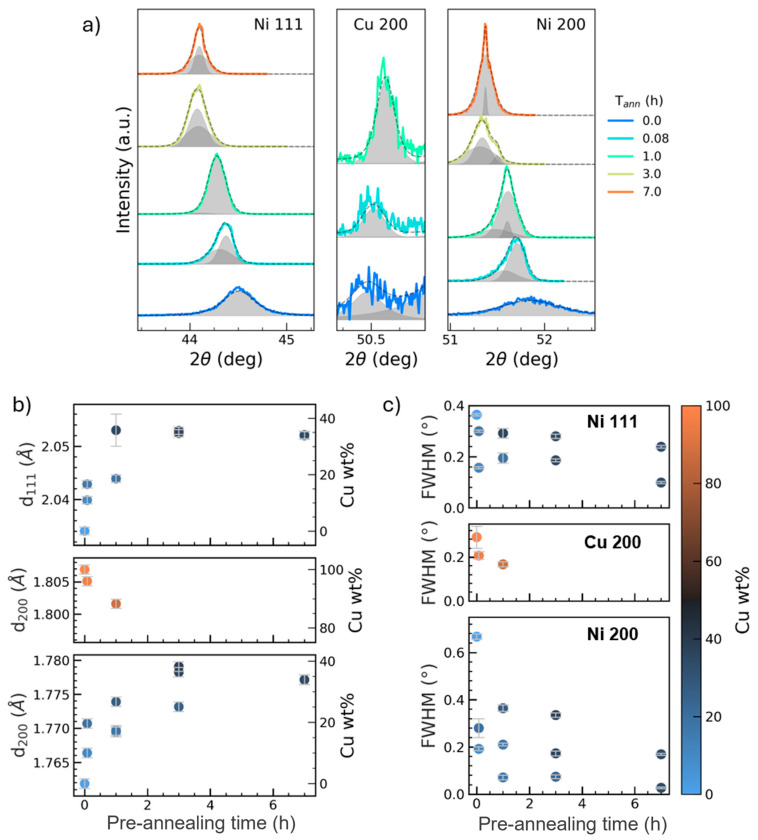
(**a**) Specular HRXRD profiles of Ni(111), Cu(200) and Ni(200) reflections with multipeak fits; individual fitted components are shown in grey. Patterns are vertically offset for clarity. (**b**) Interplanar spacings and (**c**) FWHM values extracted from the fitted peaks as a function of pre-annealing time. The corresponding estimated Cu content is reported on the secondary axis or color scale.

The compositional gradients revealed using EDS were further investigated via XRD. Specular scans collected over a wide angular range show the structural evolution upon pre-annealing (Figure S7). All detectable metal reflections are consistent with an fcc Ni-Cu structure, in agreement with the complete solid-solution behaviour of the Ni-Cu system [28]. The progressive peak shift indicates the formation of Ni-Cu alloys, while the asymmetric peak shape suggests the presence of overlapping contributions with different lattice spacings. Specular HRXRD measurements were therefore performed around selected fcc reflections, namely the Ni(111), Cu(200), and Ni(200), to quantitatively resolve the peak components and follow the alloying process (Figure 6).

The as-deposited Ni-Cu scaffold was used as the true non-annealed reference. Figure 6 also includes a short-exposure point, corresponding to a scaffold exposed only for 5 min at 1000 °C, namely **A0**, associated with the subsequent CVD process. This point is used to evaluate the early effect of high-temperature exposure on Ni-Cu alloying.

With increasing pre-annealing time, the Ni-related fcc reflections progressively shift toward lower scattering angles, corresponding to an increase in the interplanar spacing due to Cu incorporation into the Ni lattice. Conversely, the Cu-related reflection shifts toward higher scattering angles at the early stages, with Ni incorporation into the Cu lattice. At 3 h and 7 h, the pure-Cu contribution is no longer resolved in the selected HRXRD range, indicating the progressive conversion of the Cu-rich region into Ni-Cu alloyed domains.

Multipeak fitting reveals the coexistence of alloyed regions with different lattice spacings, especially at short and intermediate pre-annealing times. This behavior is consistent with interdiffusion in a porous, defect-rich electrodeposited scaffold, where grain boundaries and other defects can act as fast diffusion pathways [29]. The fitted peak positions were converted into fcc lattice parameters and used to estimate the Cu atomic fraction through a Vegard-type interpolation between pure Ni and pure Cu [30]. The values are treated as semi-quantitative because residual strain and local defects can also contribute to peak shifts. At 7 h, the fitted components converge toward an average composition of Ni_(1-x)_Cu_x_ with x ≈ 0.32 ± 0.02, although multiple fitted contributions remain visible in the FWHM analysis. The overall FWHM decrease with pre-annealing time indicates improved crystalline coherence and progressive microstrain relaxation [31].

Complementary specular/GI-XRD measurements of the Ni-Cu(311) reflection are reported in Figure S8. These data were used qualitatively to compare the lattice-spacing distribution under bulk- and surface-sensitive geometries, without assigning an absolute probing depth. The reduced geometry dependence at 7 h suggests that the remaining local compositional heterogeneity resolved using HRXRD is more uniformly distributed along the vertical direction of the porous scaffold.

### 3.3. Graphene Growth Under Fixed CVD Conditions: Multilayer and Disorder/Edge Signatures

**Figure 7:**
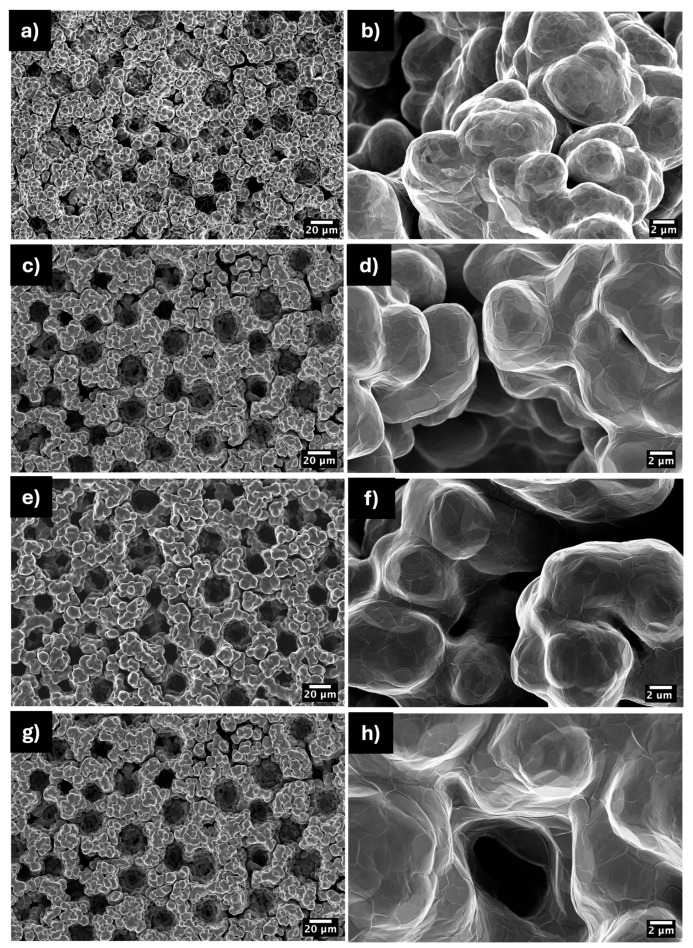
SEM images of the pre-annealed Ni-Cu scaffold after graphene growth: (**a**,**b**) **A0Gr**; (**c**,**d**) **A1Gr**; (**e**,**f**) **A3Gr**; (**g**,**h**) **A7Gr**.

Graphene was grown in situ on all **A0**–**A7** templates using an identical CVD recipe (CH_4_/H_2_, 5 min at 1000 °C, at atmospheric pressure), ensuring that differences among samples arise solely from pre-annealing time. The resulting graphene/Ni-Cu heterostructures are hereafter renamed as **A0Gr**-**A7Gr**. In Figure 7, SEM images show that the porous architecture of the scaffold was conformally coated with thin graphene layers, preserving the open pore morphology while introducing smoother surface features. High-magnification SEM images clearly showed the formation of continuous graphene sheets across the pore walls, with visible wrinkles and folds characteristic of multilayer graphene [32]. The conformal coating ensured that both external and internal surfaces of the porous framework were covered.

SEM images were taken also on the backside of **A0Gr** (Figure S9) confirming that, since both faces of the samples are exposed to CH_4_/H_2_ flux in the CVD quartz tube, graphene grows on both sides following their morphology.

Graphene growth was further investigated using Raman spectroscopy. For **A0Gr**, Raman mapping was carried out over a 10 × 10 μm^2^ area, selected on a fully focused-area under the microscope, corresponding to one crest of the 3D graphene/Ni-Cu structure. The map was acquired with a step size of 1 μm, resulting in a total of 121 probed points, with spatial resolution of ~1 μm (50× objective).

**Figure 8:**
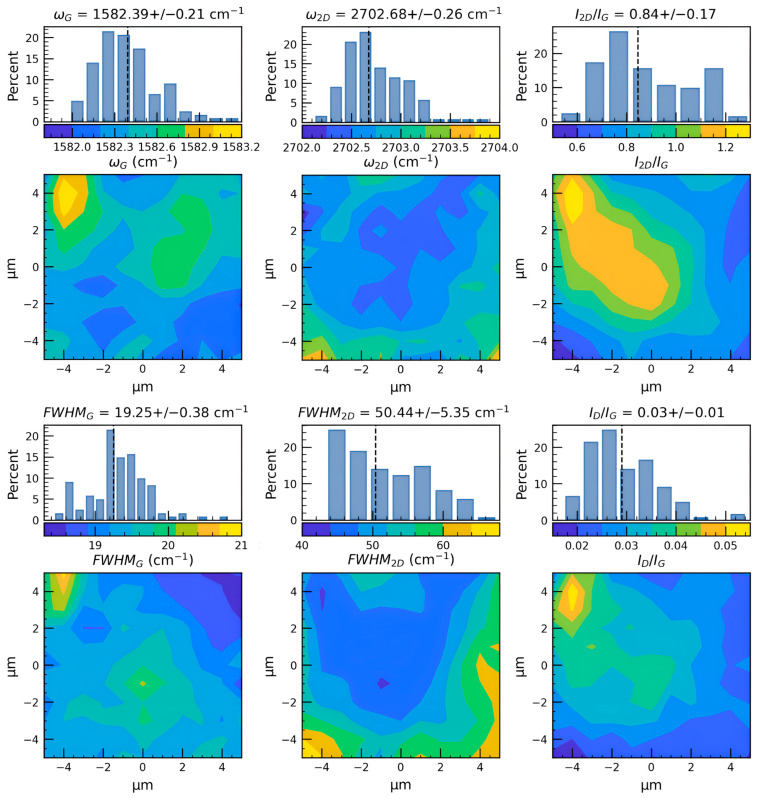
The 10 × 10 μm^2^ Raman maps and corresponding statistical distributions of the G and 2D band parameters across the scanned area of sample **A0Gr**. Parameters were extracted from fitted Raman spectra after baseline correction. For each Raman-map panel, the corresponding color scale is displayed above the map. Dashed lines in the histograms indicate the weighted mean values and corresponding standard deviation.

A detailed description of the analysis methodology employed to determine the peak positions (*ω*), full width at half maximum (FWHM), intensities (*I*), and integrated areas (*A*) of the D, G, and 2D Raman bands over the scanned area is given in Section 2.5. The corresponding maps are shown in Figure 8.

The maps reveal a certain degree of heterogeneity in the 2D band width, with FWHM values varying between 44 cm^−1^ and 65 cm^−1^. The G band width, instead, is highly reproducible across the area, showing a maximum variation in solely approximately 2.5 cm^−1^. Notably, as visible from the statistical distribution of I_2D_/I_G_, in more than 35% of the probed points, the 2D band intensity is comparable to or higher than that of the G band (*I*_2D_/*I*_G_ > 0.9). For these spectra the 2D band appears very symmetric, similarly to spectrum reported in Figure S18. Additionally, the D band is extremely weak, *I*_D_/*I*_G_ < 0.04 in more than the 90% of the scanned area, indicating a low defect density within the graphene lattice. Overall, these results point to a graphene network with a low defect density, although exhibiting structural discontinuities. The Raman signatures suggest a locally varying thickness within the two-to-four-layer regime when compared with those of planar graphene reported in the literature [33,34,35]. These observations are also consistent with the findings in Huang et al., where a similar graphene architecture was reported [23].

For the pre-annealed samples (**A1Gr**, **A3Gr** and **A7Gr**), Raman spectra on five random areas of the outer surfaces, in contrast to what found for **A0Gr**, reveal highly homogeneity. The 2D band is broader and asymmetric, while the ratio *I*_2D_/*I*_G_ is ≤ 0.6 across all spectra, suggesting a more homogeneous and thicker material (ca. 4–5 layers) [36]. The defective-related D band is still very weak, confirming the good quality of the graphene film. The main spectral features are summarized in Table S1. Since the high reproducibility of data, only one representative spectrum for each sample is plotted in Figure S18 for direct comparison.

XRD measurements were also performed on the graphene-coated Ni-Cu scaffolds before etching. However, the diffraction signal was dominated by the metallic template, and graphenic or graphite-like reflections could not be unambiguously resolved. Therefore, XRD was not used to validate the Raman assignment before template removal. The structural organization of the freestanding graphenic network was assessed via XRD only after etching of the Ni-Cu scaffold, as discussed in Section 3.4.

### 3.4. Template Removal and Freestanding 3D Graphene: Morphology Retention and Structural Properties

**Figure 9:**
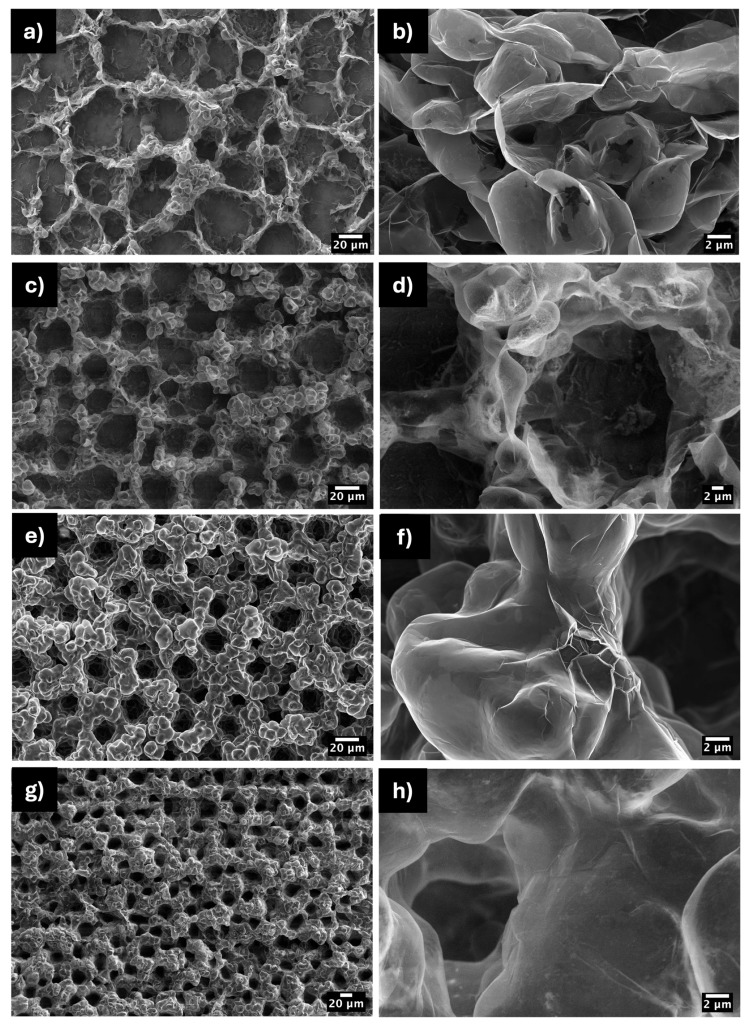
SEM images of the freestanding graphene foam after Ni-Cu template etching of samples showing well-preserved porous architecture without collapse: (**a**,**b**) **Gr0**; (**c**,**d**) **Gr1**; (**e**,**f**) **Gr3**; (**g**,**h**) **Gr7**.

By using the wet etching procedure detailed in the synthesis section, the Ni-Cu alloy scaffold was fully removed from **A0Gr–A7Gr** without employing any polymeric support layer, such as PMMA. The resulting freestanding 3D graphene architectures, hereafter denoted as **Gr0–Gr7**, were subsequently sectioned into two pieces and transferred onto glass substrates, enabling exposure of both the front and back surfaces. Post-etch SEM in Figure 9 reveals a strong dependence on pre-annealing time: **Gr0** and **Gr1** do not preserve a compact, clearly porous architecture after etching, whereas **Gr3** yields a stable, freestanding 3D porous graphene network. **Gr7** shows similar post-etch morphology and overall results to **Gr3**, indicating that structural reinforcement and porous architecture retention of the graphene network reach a plateau by ~3 h.

Cross-sectional and backside SEM images reveal a thin, continuous, and relatively flat graphene-like layer at the bottom of the etched porous network (Figures S10 and S11), rather than a replica of the highly discontinuous Ni-Cu backside, with a total thickness around 40 µm. The precise origin of this relatively flat graphenic layer cannot be determined unambiguously. It is tentatively attributed to graphenic material formed in the lower regions of the porous scaffold during CVD and subsequently exposed, transferred, flattened, or restacked during metal removal and drying. The observation of graphene-like fragments floating during etching suggests that redistribution of carbon sheets may also contribute to the final morphology.

**Figure 10:**
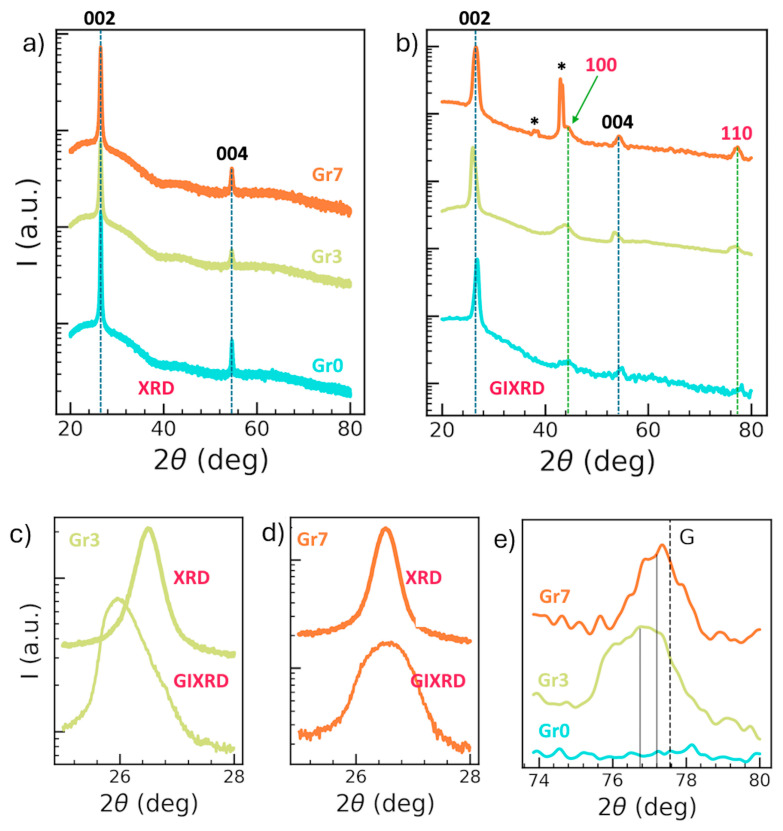
(**a**,**b**) Specular and GI-XRD patterns collected after removal of the Ni-Cu template. The 002, 004, 100, and 110 reflections are indicated; the asterisk marks the contribution from the glue used for sample mounting. (**c**,**d**) Comparison of the 002 reflection in specular and GI geometries for **Gr3** and **Gr7**. (**e**) GI-XRD profiles of the 110 reflection used to evaluate the in-plane lattice distortion. Gray lines indicate the evolution of peak position.

XRD measurements were performed after removal of the Ni-Cu scaffold, when the graphenic signal could be isolated from the dominant diffraction of the metallic template. The corresponding patterns are reported in Figure 10. In specular geometry, the etched foams show the 002 reflection, together with the weaker 004 harmonic, indicating the presence of vertically stacked graphenic layers. In GI-XRD geometry, additional reflections associated with the in-plane hexagonal lattice become visible, including the 100 and 110 peaks. The region around the 100 reflection is affected by the glue used for sample mounting; therefore, the 110 reflection was used to evaluate the in-plane lattice distortion.

The **Gr0** sample shows a weak and poorly defined in-plane hexagonal-lattice signal. This behaviour is attributed to the fragility of the graphenic network formed when no template pre-annealing is performed. After metal removal, the structure collapses and the graphenic sheets flatten onto the bottom layers. As a result, the diffraction signal is dominated by more oriented stacked layers, whereas the in-plane reflections expected from a self-supporting, polycrystalline 3D network of graphenic walls are strongly suppressed. This interpretation is consistent with the SEM evidence of structural collapse after etching. The quantitative comparison was therefore focused mainly on the self-supporting **Gr3** and **Gr7** foams.

From the specular 002 reflection, **Gr3** and **Gr7** show the same interlayer spacings (d002 = 3.364(2) Å and 3.361(2) Å, respectively), indicating comparable average stacking along the out-of-plane direction. These values are slightly larger than the interlayer spacing of ideal graphite (~3.354 Å), suggesting a certain degree of turbostratic disorder or weakened interlayer coupling. However, the GI-XRD measurements, which are more sensitive to the outer regions of the 3D structure, reveal a larger interlayer spacing for **Gr3** compared to **Gr7**. This increase in d002 in GI geometry is consistent with a reduced interlayer coupling and a more turbostratic stacking of the graphenic layers, in agreement with the Raman signatures, reported below, of weaker interlayer interaction and increased structural disorder. For **Gr7**, the GI-derived interlayer spacing approaches the specular value, suggesting a more uniform and better-coupled stacking arrangement after longer pre-annealing of the Ni-Cu template.

The in-plane structure follows the same trend. From the 110 reflection, the estimated in-plane tensile lattice strain along the ⟨110⟩ direction decreases from about 0.9% for **Gr3** to about 0.4% for **Gr7**, while the FWHM decreases from 1.74(8)° to 1.35(7)°. Since graphenic reflections could not be isolated before etching, this strain must be interpreted as a residual post-etch strain of the freestanding graphene foam, rather than a direct measurement of the strain state during CVD growth on the metallic template. The decrease observed from **Gr3** to **Gr7** indicates that longer template pre-annealing reduces the strain retained in the final freestanding network after metal removal.

Raman mapping was performed on all etched samples (**Gr0**, **Gr1**, **Gr3**, **Gr7**) under the same experimental conditions used for the unannealed samples prior to etching (**A0Gr**). The spectral maps obtained after baseline subtraction and peak fitting (see Figure S12) are reported for **Gr0–Gr7** in Figures S13–S17, respectively. A statistical comparison of the weighted average Raman parameters before and after etching, mainly summarized in Table 1 and Table S1, shows that removal of the metallic template influences the spectroscopic response of the graphenic network. Specifically, the following observation can be made:

- Degradation after etching. After substrate removal, **Gr0** retains a relatively sharp G band and a detectable 2D band, superimposed on a broad contribution extending across the D–G spectral region. The baseline correction and multicomponent analysis of this spectral envelope are illustrated in Figure S12, while the comparison with the spectrum collected before etching is reported in Figure S18. The D and D′ bands are conventionally associated with defect-activated Raman scattering in graphenic sp^2^ structures [37]. However, broad multicomponent envelopes involving contributions in the D, F, D″, G, and D′ spectral regions have also been reported for graphene-derived disordered and two-dimensional aromatic amorphous sp^2^ carbon [38]. Therefore, particularly for **Gr0**, the fitted contribution near 1350 cm^−1^ cannot be interpreted as an isolated D mode of otherwise crystalline graphene. Rather, the spectrum is consistent with the coexistence of preserved graphenic or graphite-like domains, responsible for the relatively sharp G and detectable 2D bands, and a strongly disordered or locally amorphous sp^2^-carbon contribution underlying the broad D-G envelope. The pronounced post-etch spectral modification is consistent with the severe collapse of the three-dimensional architecture observed via SEM in Figure 9. In the pre-annealed samples, the disorder-related spectral contributions are substantially weaker, with the lowest overall contribution observed for **Gr7**. This trend is illustrated in Figure S18, which reports representative Raman spectra collected before and after etching for all samples. We note that, in view of the very low intensity and partial overlap of the disorder-related components in the pre-annealed samples, Raman spectroscopy does not allow a reliable quantitative assessment of the graphene defect density. Therefore, these fitted components are not interpreted here in terms of specific defect types or defect concentrations, but rather as qualitative indicators of the structural degradation induced by the etching process. Within this framework, an operational threshold-based analysis was used to compare the occurrence of disorder-related spectral contributions across Raman maps acquired under identical experimental and fitting conditions. The combined fitted area associated with the D′, D″ and F components was compared with that of the G band, and three arbitrary thresholds of 0.5, 1 and 1.5 were considered. For each threshold, the fraction of analysed map points exceeding the selected value was calculated. The threshold-based disorder-related map fraction reported in Table 1 corresponds to the average of the three estimates, while the reported dispersion describes their threshold-to-threshold variability. This parameter is intended only as an empirical descriptor for relative comparison among the etched samples. It does not represent a direct geometrical measurement of degraded area or an absolute graphene defect density, because each Raman spectrum averages the graphenic material contained within the laser-probed volume. Moreover, because measurements were intentionally collected on fully focused crest regions, the resulting values refer specifically to the analysed portions of the graphenic walls and do not include the complete distribution of pore-edge contributions. Despite these limitations, the same acquisition, processing, fitting, and threshold criteria were applied to all samples. The analysis therefore provides a consistent internal comparison and reveals a marked decrease in the occurrence of disorder-related spectral contributions from **Gr0** to the pre-annealed samples, with the lowest value observed for **Gr7**. Together with the SEM observations, this trend supports improved preservation of the graphenic network during metal removal and drying after template pre-annealing.
- Probed thickness. Before etching, the main Raman thickness difference is observed between **A0Gr** and the pre-annealed series. **A0Gr** exhibits a locally heterogeneous few-layer-like response, whereas **A1Gr**, **A3Gr**, and **A7Gr** show broadly comparable multilayer-like signatures. The greater local thickness of the pre-annealed graphenic coatings may contribute to their improved resistance to metal removal and drying. However, because **Gr1** does not preserve the porous architecture whereas **Gr3** and **Gr7** do, graphenic thickness alone cannot explain the observed post-etch stability threshold. For **Gr0** the I_2D_/I_G_ ratio decreases by nearly a factor of two, while the band undergoes substantial broadening, with the FWHM increasing from approximately 50 cm^−1^ to 80 cm^−1^. This behavior is consistent with an increase in the effective thickness of the graphenic material, most likely resulting from structural collapse after etching and the consequent probing of a larger number of stacked graphene layers. These findings are in good agreement with the results obtained from XRD analysis. In contrast, the samples pre-annealed for 3 h and 7 h exhibit I_2D_/I_G_ ratios and 2D-band FWHM values comparable to those measured prior to etching, consistently with the preservation of the graphenic network during template removal.
- Strain. For all the samples, the average position of the 2D band reported in Table 1 shifts to lower wavenumbers after etching. It should be noted that these values were calculated as the area-weighted average position of the four fitted components (see Supporting Information). This downshift is evident in the spectra as an increased asymmetry of the 2D band, arising from the larger contribution of the lower-wavenumber components (Figures S12 and S18). The observed behavior is consistent with the development of tensile strain in the freestanding foam. The shift is accompanied by a broadening of the 2D band, while the G band is less affected. Therefore, the post-etch Raman response suggests that tensile strain is introduced or amplified during metal removal, in agreement with the residual in-plane strain measured via XRD on the etched foams.

#### 3.4.1. Electrical Properties

The surface sheet resistance for the 3D graphene foams **Gr0**–**Gr7** was determined using the four-point probe method, a standard and reliable technique that minimizes the influence of contact resistance. The foams, approximately 25–40 µm thick and consisting of few-layer graphene, were probed on fifteen different regions of their front surface to ensure statistical reliability. Despite the structural degradation observed in **Gr0** and **Gr1** via SEM and Raman analyses, all samples exhibited comparable sheet resistance values, ranging between 10 and 20 Ω/□. Because the four-point measurements probe the complete transferred carbon specimen, a contribution from the relatively flat graphenic layer observed at the bottom of the etched network cannot be excluded. The measured sheet resistance may therefore include parallel current pathways through both the porous three-dimensional network and the flatter graphenic base. Accordingly, the reported values should be regarded as effective sheet resistances of the complete freestanding carbon structure rather than as the isolated electrical response of the porous network. The low sheet resistance values indicate that continuous conductive pathways persist, even in the partially collapsed samples, and are comparable to values reported for similarly structured graphene foams [39]. These qualities further support the suitability of the material for a wide range of applications, including its use as a three-dimensional conductive platform for the deposition of electrocatalysts in HER and CO_2_RR systems.

#### 3.4.2. Optical Properties

The optical properties of the 3D graphene foam scaffolds were characterized to evaluate the light-harvesting potential for multiple applications including photoelectrochemical (PEC) applications, including hydrogen evolution reaction (HER) and CO_2_ reduction reaction (CO_2_RR). Ultraviolet–visible–near-infrared (UV–Vis–NIR) spectroscopy combined with an integrating sphere was employed to measure the total reflectance (*R*) and transmittance (*T*) over the 300–1000 nm spectral range under both front- and back-side illumination of **Gr0**–**Gr7** graphene foams.

As shown in Figure S19, all graphene foam samples exhibit extremely low transmittance throughout the spectral range investigated, with *T* values ranging from approximately 0.5% to 4.5%. With a thickness of 25~40 μm, the porous graphene network is therefore essentially opaque to incident light, indicating that nearly all incoming photons are either absorbed within the structure or reflected from its surface.

From the integrating-sphere measurements, the absorptance (*A*) of the foam, calculated as *A* = 1 − *R*–*T*, stays in the range of roughly 75–90% across the visible spectrum, similarly to what reported in [40] for standard graphene foam. This high absorptance qualifies graphene foams as a “broadband absorbers,” consistent with the known optical behavior of carbon-based materials. For two-dimensional graphene, a single atomic layer absorbs only ~2.3% of incident visible light and remains highly transparent [41]. The dramatic enhancement in photon capture observed here therefore originates primarily from stacking multiple graphene layers forming 3D networks. The high absorptance can be attributed to the synergistic combination of few-layer graphene walls, and high porosity. Light entering the foam experiences multiple scattering and repeated internal reflections, which increase the effective optical path length and substantially enhance the probability of photon absorption. Similar photon-trapping mechanisms have been reported for three-dimensional graphene aerogels and hierarchical graphene-based structures developed for solar-energy conversion and photocatalytic applications [1,2,3].

Interestingly, a pronounced asymmetry in total reflectance was observed depending on the illumination direction. When illuminated from the porous “front” side, the foam reflects only about *R* ≈ 10–12% of incident light. In contrast, illumination from the “back” flat side yields a higher reflectance, *R* ≈ 19–22%. This difference can be directly correlated with the morphological characteristics of the two surfaces. In the front porous side, light entering can undergo multiple internal reflections within pores, effectively getting “trapped,” whereas the opposite flat side retains a smoother graphene skin, increasing reflectance before it penetrates deeply.

Overall, the optical response of our multilayer 3D graphene foams—characterized by low front reflectance (~10%), negligible transmittance (<5%), and high absorptance (75–90%)—is highly desirable for PEC electrodes. These materials effectively behave as photon-trapping scaffolds, funneling illumination into the electrode where it can be utilized for driving reactions. These characteristics closely match those reported for other light-trapping carbon foams and hierarchical graphene structures designed for solar fuel generation [42]. Coupled with its chemical stability under a wide pH range, and large internal surface, this foam can serve as an efficient conductive, light-absorbing support for catalysts in HER and CO_2_RR systems.

## 4. Discussion

The results identify the pre-annealing-driven evolution of the Ni-Cu template as the main processing variable associated with the formation and stability of the freestanding graphene foam. Since the CVD growth conditions were kept unchanged throughout the series, the differences observed after metal removal can be primarily related to the compositional and microstructural evolution of the metallic scaffold.

During pre-annealing, Cu diffuses from the underlying foil into the porous electrodeposited Ni network while the macroporous architecture is retained. This process differs from diffusion through a compact planar film because the electrodeposited scaffold contains a high density of surfaces, grain boundaries, and structural defects that can provide fast diffusion pathways. This explains the measurable Cu incorporation after the first high-temperature exposure and the pronounced compositional evolution observed within the first 3 h. EDS shows that most Cu incorporation occurs over this interval, whereas HRXRD reveals that alloying proceeds through several local Ni-Cu compositions rather than through the immediate formation of a homogeneous solid solution. Longer pre-annealing promotes compositional redistribution and microstructural relaxation, as indicated by the convergence and narrowing of the fitted diffraction components. Nevertheless, local heterogeneity remains at 7 h, although the specular/GI-XRD comparison indicates that it becomes less dependent on vertical position within the scaffold. The long-pre-annealed template is therefore better described as a compositionally stabilized and relaxed, but still locally heterogeneous, Ni-Cu alloy.

This structural evolution modifies the catalytic environment for graphene growth. Ni-rich regions can support carbon dissolution at the CVD temperature followed by segregation or precipitation during cooling, favoring multilayer graphenic stacking. In more Cu-rich regions, the lower carbon solubility shifts the balance toward a more surface-mediated growth regime. Grain boundaries, residual defects, and the local surface morphology can further influence hydrocarbon decomposition, carbon diffusion, and nucleation. Progressive Ni-Cu alloying is therefore expected to reduce the spatial contrast between these growth environments and to favor a more uniformly connected graphenic coating along the pore walls.

The post-etch morphology is consistent with this interpretation. Graphenic coatings are formed under all investigated conditions, but **Gr0** and **Gr1** do not preserve a well-defined porous architecture after metal removal. Their carbon networks are therefore inferred to be insufficiently continuous or structurally interconnected to withstand the stresses associated with wet etching, capillary forces, and drying. By contrast, **Gr3** and **Gr7** retain the porous structure, consistent with the formation of an interconnected multilayer graphenic shell capable of supporting itself after removal of the metallic scaffold. The XRD response of collapsed **Gr0**, dominated by oriented stacking with strongly suppressed in-plane reflections, further supports the flattening of the graphenic walls onto the substrate rather than the retention of a polycrystalline three-dimensional network. Taken together, the SEM and XRD results establish a clear processing–structure correlation, although they do not isolate a single microscopic mechanism; local graphenic thickness, coating continuity, network connectivity, and stacking organization may contribute jointly to post-etch morphological preservation.

Approximately 3 h can therefore be identified as a practical threshold under the present experimental conditions. By this time, most Cu incorporation has occurred, the pure-Cu contribution is no longer resolved, and the alloy distribution has become sufficiently stabilized across the scaffold. **Gr3** is also the first sample in the investigated series to preserve the freestanding porous architecture after etching. Extending the pre-annealing time to 7 h produces further local improvements, including a more uniform stacking arrangement, fewer defect-related Raman contributions, and lower residual strain, but does not result in a qualitatively different macroscopic foam morphology. The 3 h condition should therefore not be regarded as the time required to reach complete chemical equilibrium, but as the point at which alloy evolution and graphenic-network connectivity become sufficient to ensure post-etch structural stability.

The Raman and XRD results after etching provide complementary information on the structural response of the freestanding network. Template removal shifts the Raman 2D band toward lower wavenumbers, compatibly with tensile strain introduced or amplified during metal dissolution, mechanical release, wet processing, and drying. Consistently, the strain derived from the XRD 110 reflection must be interpreted as residual post-etch strain, because graphenic reflections could not be isolated before removal of the metallic scaffold. Its lower value in **Gr7** than in **Gr3** therefore indicates that longer template pre-annealing leads to a network that retains less strain after etching, rather than demonstrating a direct reduction in graphene strain during CVD growth. Together with the lower disorder-related Raman response and reduced stacking inhomogeneity, this result indicates improved structural organization of the final freestanding foam.

The electrical and optical measurements confirm the functional relevance of the resulting architectures. All etched samples retain low sheet resistance, showing that electrical connectivity can persist even after partial morphological collapse. However, the preserved three-dimensional structure of **Gr3** and **Gr7** is essential for maintaining accessible surface area, structural integrity, and pathways for mass transport. Their multilayer graphenic walls and hierarchical porosity also produce negligible transmittance and strong broadband absorptance, while the lower reflectance from the porous front side confirms the role of multiple scattering and internal reflection in photon trapping. Overall, controlled pre-annealing links Ni-Cu alloy formation, graphene-growth conditions, post-etch structural stability, and functional response, with approximately 3 h providing the most relevant transition toward a robust freestanding graphene foam.

## 5. Conclusions

Freestanding three-dimensional graphene foams were successfully fabricated by CVD growth on porous electrodeposited Ni-Cu scaffolds followed by template removal. Optimization of the electrodeposition process enabled the reproducible formation of interconnected porous structures, while high-temperature pre-annealing promoted Ni-Cu alloying and compositional stabilization without compromising the macroporous architecture.

The evolution of the Ni-Cu template strongly influences graphene growth and the stability of the resulting freestanding network. While low-defect graphene can be obtained even on non-alloyed templates, only scaffolds pre-annealed for at least 3 h generate graphenic shells sufficiently continuous and interconnected to retain the three-dimensional architecture after metal removal. Raman and XRD analyses indicate that increasing pre-annealing time improves structural organization of the graphene network, reducing strain and stacking disorder, ultimately minimizing degradation after the removal of the metal scaffold.

The optimized foams combine well-preserved 3D morphology with low sheet resistance, negligible transmittance, and strong broadband visible-light absorption, highlighting the benefits of the hierarchical porous architecture for charge transport and photon trapping. These findings demonstrate that controlled Ni-Cu alloying is a critical parameter for tailoring both graphene growth and the stability of the resulting freestanding network. By promoting the formation of continuous and interconnected graphenic shells, alloyed templates enable the fabrication of lightweight, conductive, and structurally resilient graphene foams with significant potential for electrocatalytic and photoelectrochemical energy-conversion applications.

While further efforts are needed to improve handling of the ultrathin graphene framework, evaluate long-term operational durability, quantify device-level performance, and extend the process to larger areas, the present work establishes a scalable materials-design strategy based on alloy-assisted template engineering. The ability to tune graphene quality and foam preservation through controlled Ni-Cu interdiffusion provides a versatile foundation for the development of next-generation three-dimensional graphene electrodes combining high surface area, efficient charge transport, and enhanced light-management capabilities.

## Figures and Tables

**Table 1:** Weighted mean Raman parameters and corresponding weighted standard deviations for **A0Gr** and **Gr0–Gr7** obtained from Raman maps. The threshold-based disorder-related Raman-map fraction corresponds to the mean percentage of analysed map points exceeding the three predefined spectral thresholds (0.5, 1, and 1.5); the reported uncertainty is the standard error of the three threshold-derived estimates. This operational parameter is used only for relative comparison among the etched samples and does not represent a direct geometrical measurement of degraded area or an absolute graphene defect density.

	A0Gr	Gr0	Gr1	Gr3	Gr7
*ω*_D_ (cm^−1^)	1354.07 ± 0.88	1370.09 ± 11.54	1356.40 ± 9.68	1360.84 ± 6.73	1357.45 ± 4.55
*ω*_G_ (cm^−1^)	1582.39 ± 0.21	1581.23 ± 0.15	1580.43 ± 0.30	1580.80 ± 0.15	1580.52 ± 0.16
*ω*_2D_ (cm^−1^)	2702.68 ± 0.26	2695.40 ± 1.67	2700.28 ± 1.97	2701.91 ± 2.42	2702.51 ± 0.79
*I*_2D_/*I*_G_	0.84 ± 0.17	0.47 ± 0.03	0.45 ± 0.04	0.44 ± 0.03	0.47 ± 0.02
*A*_2D_/*A*_G_	1.81 ± 0.22	1.06 ± 0.21	1.18 ± 0.15	1.16 ± 0.12	1.26 ± 0.06
FWHM_G_ (cm^−1^)	19.25 ± 0.38	24.76 ± 2.31	20.87 ± 2.25	22.46 ± 1.91	20.12 ± 0.80
FWHM_2D_ (cm^−1^)	50.44 ± 5.35	79.76 ± 2.34	73.68 ± 3.06	78.08 ± 2.25	75.57 ± 1.61
Threshold-based disorder-related Raman-map fraction (%)	NA	99.2 ± 0.8	46 ± 8	34 ± 9	3 ± 3

## Data Availability

The original contributions presented in this study are included in the article/Supplementary Material. Further inquiries can be directed to the corresponding authors.

## Supplementary Materials

The following supporting information can be downloaded at: https://www.mdpi.com/article/10.3390/nano16150950/s1, Figure S1: SEM images of the Ni-Cu scaffolds fabricated at I = 1 A (0.25 A/cm2), 1.6 A (0.40 A/cm2), 2.4 A (0.60 A/cm2) after different deposition times.; Figure S2: Cross-sectional view SEM images of the Ni-Cu scaffolds fabricated at different electrodeposition currents: (a) *I* = 1 A (0.25 A/cm^2^), (b) *I* = 1.6 A (0.40 A/cm^2^), (c) *I* = 2.4 A (0.60 A/cm^2^). While the thickness of the electrodeposited Ni layer is uniform on the front side, the backside exhibits a strong thickness gradient, ranging from approximately 40 µm near the edges of the Cu foil to only a few nanometers in the central region.; Figure S3: Image analysis routine: (a) portion of a SEM raw image, (b) image whose contrast and brightness have been improved using the local contrast enhancement (CLAHE) algorithm, (c) image cleaned from nanoscale pores with a gaussian blur, (d) binarized image using a local threshold and Otsu algorithm, (e) image treated with a cycle of morphological transformations (closure/erosion/dilation) al-ternated with the watershed algorithm to separate close pores and smooth their profiles (f) original image overlapped with the contours identified on the pores.; Figure S4: Analysis of pore size distribution: (a) combined pore size distribution for the *I* = 2.4 A sample, presenting a polydisperse distribution of sizes. On the right are shown the maps of the Sauter radius R32 across multiple portions of the samples: (b) the *I* = 2 A batches A, B, C; (c) the *I* = 2.4 A sample.; Figure S5: SEM images of the Ni-Cu scaffolds, fabricated with *I* = 2 A, after different pre-annealing times: (a,b) 1 h (**A0**); (c,d) 3 h (**A3**); (e,f) 7 h (**A7**).; Figure S6: EDS Maps of the Ni-Cu scaffolds fabricated with *I* = 2 A after different pre-annealing times: (a) no pre-annealing (A0); (b) 1 h (**A1**); (c) 3 h (**A3**); and (d) 7 h (**A7**).; Figure S7: Specular XRD patterns of Ni–Cu scaffolds as a function of pre-annealing time. Patterns are vertically offset for clarity. The main FCC Ni and Cu reflections are labeled.; Figure S8: (a–c) Comparison of the Ni-Cu (311) reflection profiles collected in specular geometry and at GI angles of 10° and 4° vs pre-annealing times. (d,e) Interplanar spacing and FWHM values extracted from the 311 reflection as a function of pre-annealing time. The corresponding estimated Cu content is reported on the secondary axis or color scale (f).; Figure S9: SEM images of the backside of **A0Gr** sample.; Figure S10: SEM images at different enlargement of the selfstanding Graphene freestanding graphene foams: (a,b) **Gr0**; (c,d) **Gr1**; (e,f) **Gr3**; (g,h) **Gr7**.; Figure S11: (a) top and (b) cross-sectional view SEM images freestanding graphene foam **Gr7** from its backside.; Figure S12: Example of Raman spectra for **Gr0**. (a) Photoluminescence baseline fitting and subtraction from raw data. The baseline has been fitted to the data in the ranges [1800, 2400]–[2800, 2850] cm^−1^, and interpolated/extrapolated to the regions containing peaks. (b) Multiple peak fitting on the treated data, in units of *I*_2D_. The individual components are highlighted by colored lines, and the full fit is represented by a dashed line. (c) Residuals between the data and the fit.; Figure S13: 10 × 10 μm^2^ Raman maps and corresponding statistical distributions of the G and 2D band parameters across the scanned area of sample **Gr0**. The parameters were obtained from baseline-corrected Raman spectra and extracted through peak fitting. Dashed lines in the histograms denote the weighted mean values, which are reported above each plot together with their corresponding standard deviations.; Figure S14: 10 × 10 μm^2^ Raman maps and corresponding statistical distributions of the G and 2D band parameters across the scanned area of sample **Gr1**. The parameters were obtained from baseline-corrected Raman spectra and extracted through peak fitting. Dashed lines in the histograms denote the weighted mean values, which are reported above each plot together with their corresponding standard deviations.; Figure S15: 10 × 10 μm^2^ Raman maps and corresponding statistical distributions of the G and 2D band parameters across the scanned area of sample **Gr3**. The parameters were obtained from baseline-corrected Raman spectra and extracted through peak fitting. Dashed lines in the histograms denote the weighted mean values, which are reported above each plot together with their corresponding standard deviations.; Figure S16: 10 × 10 μm^2^ Raman maps and corresponding statistical distributions of the G and 2D band parameters across the scanned area of sample **Gr7**. The parameters were obtained from baseline-corrected Raman spectra and extracted through peak fitting. Dashed lines in the histograms denote the weighted mean values, which are reported above each plot together with their corresponding standard deviations.; Figure S17: 10 × 10 μm^2^ Raman maps and corresponding statistical distributions of the *A*_D_/*A*_G_ ratio across the scanned area of the samples (from top left to bottom right): **A0Gr**, **Gr0**, **Gr1**, **Gr3**, **Gr7**. The parameters were obtained from baseline-corrected Raman spectra and extracted through peak fitting. Dashed lines in the histograms denote the weighted mean values, which are reported above each plot together with their corresponding standard deviations.; Figure S18: Representative Raman spectra for each sample before and after wet etching of the Ni-Cu scaffold. The spectra from maps have been selected to exhibit with I_2D_/I_G_ values comparable to the weighted average values reported in Table 1 and Table S1. Data are normalized to 2D peak intensity and arbitrarily shifted for each sample to allow a clearer comparison. The main peak components are labelled; the asterisk marks a peak attributed to contaminants on the sample.; Figure S19: From top to bottom, UV–Vis–NIR Transmittance, Reflectance and Absorptance spectra measured from the front (left) and back (right) sides for the 3D graphene foam. The high noise level is due to the use of the integrating sphere, which decreases the intensity of the light reaching the detector. Consequently, the signal-to-noise ratio is reduced, making the measurements more sensitive to noise.; Table S1: Weighted average of the spectral parameter with their weighted standard deviations for **A1Gr**-**A7Gr**, estimated from five random areas each. References [37,43,44,45] are cited in the supplementary materials.

## Abbreviations

The following abbreviations are used in this manuscript:

CVD	Chemical Vapor Deposition
SEM	Scanning Electron Microscopy
EDS	Energy-Dispersive X-ray Spectroscopy
FWHM	Full Width at Half Maximum
XRD	X-Ray Diffraction
GI-XRD	Grazing Incidence X-Ray Diffraction
HRXRD	High Resolution X-Ray Diffraction
