# Supplementary material for: Freestanding 3D Multilayer Graphene Foams from Nanotextured Ni-Cu Templates
Source: Nanomaterials (Basel). 2026 Aug 1;16(15):950. doi: 10.3390/nano16150950 (PMC13468058; doi:10.3390/nano16150950)
Supplement: Supplementary file 1 [file nanomaterials-16-00950-s001.zip › nanomaterials-4454784-supplementary.pdf]

nanomaterials

Supplementary Materials: Freestanding 3D Multilayer Graphene Foams from Nanotextured Ni-Cu Templates

Jaimon Chonedan Johnson [1,2,†], Nicolò Galvani [2,†], Piera Maccagnani [2], Alessandro Surpi [2], Nicola Gilli [2], Rita Rizzoli [2], Alessandro Gradone [2,*], Giulia Lorusso [2,*], Fabiola Liscio [2] and Vittorio Morandi [3]

[1] Dipartimento di Scienza Applicata e Tecnologia (DISAT), Politecnico di Torino, Corso Duca degli Abruzzi, 24, 10129 Torino, Italy; jaimon.chonedan@polito.it
[2] Istituto per lo Studio dei Materiali Nanostrutturati-Consiglio Nazionale delle Ricerche (ISMN-CNR Bologna), Via Piero Gobetti 101, 40129 Bologna, Italy; nicologalvani@cnr.it (N.G.); alessandro.surpi@cnr.it (A.S.); nicola.gilli@cnr.it (N.G.); fabiola.liscio@cnr.it (F.L.)
[3] Istituto per lo Studio dei Materiali Nanostrutturati-Consiglio Nazionale delle Ricerche (ISMN-CNR), Strada Provinciale 35d, n. 9, 00010 Montelibretti, Italy; vittorio.morandi@cnr.it
* Correspondence: alessandro.gradone@cnr.it (A.G.); giulia.lorusso@cnr.it (G.L.)
† These authors contributed equally to this work.

Index:

Electrodeposition conditions vs morphology pag.2

Porosity evaluation pag.3

Alloying vs pre-annealing time pag.5

Supplementary XRD analysis of Ni-Cu alloy formation pag.6

Specular/GI-XRD analysis of the Ni-Cu(311) reflection pag.7

Graphene on Ni-Cu scaffold.. pag.8

3D Etched Graphene pag.10

Multi-Peak fitting procedure for Raman spectra.. pag.11

Raman results pag.13

Optical Measurements pag.19

 https://doi.org/10.3390/nano16150950

Electrodeposition conditions vs morphology

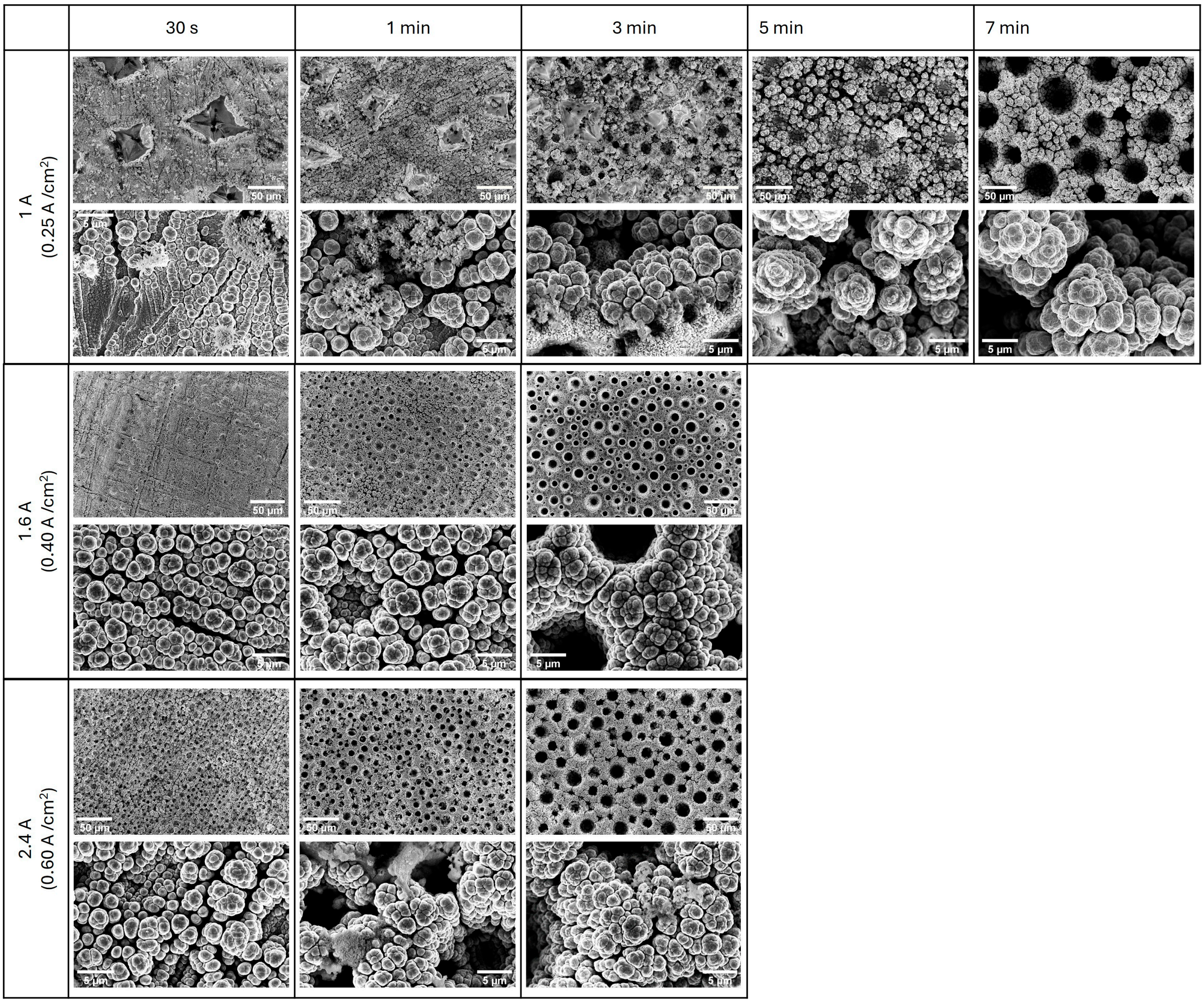

Figure S1. SEM images of the Ni-Cu scaffolds fabricated at *I* = 1 A (0.25 A/cm^2), 1.6 A (0.40 A/cm^2), 2.4 A (0.60 A/cm^2) after different deposition times.

https://doi.org/10.3390/nano16150950

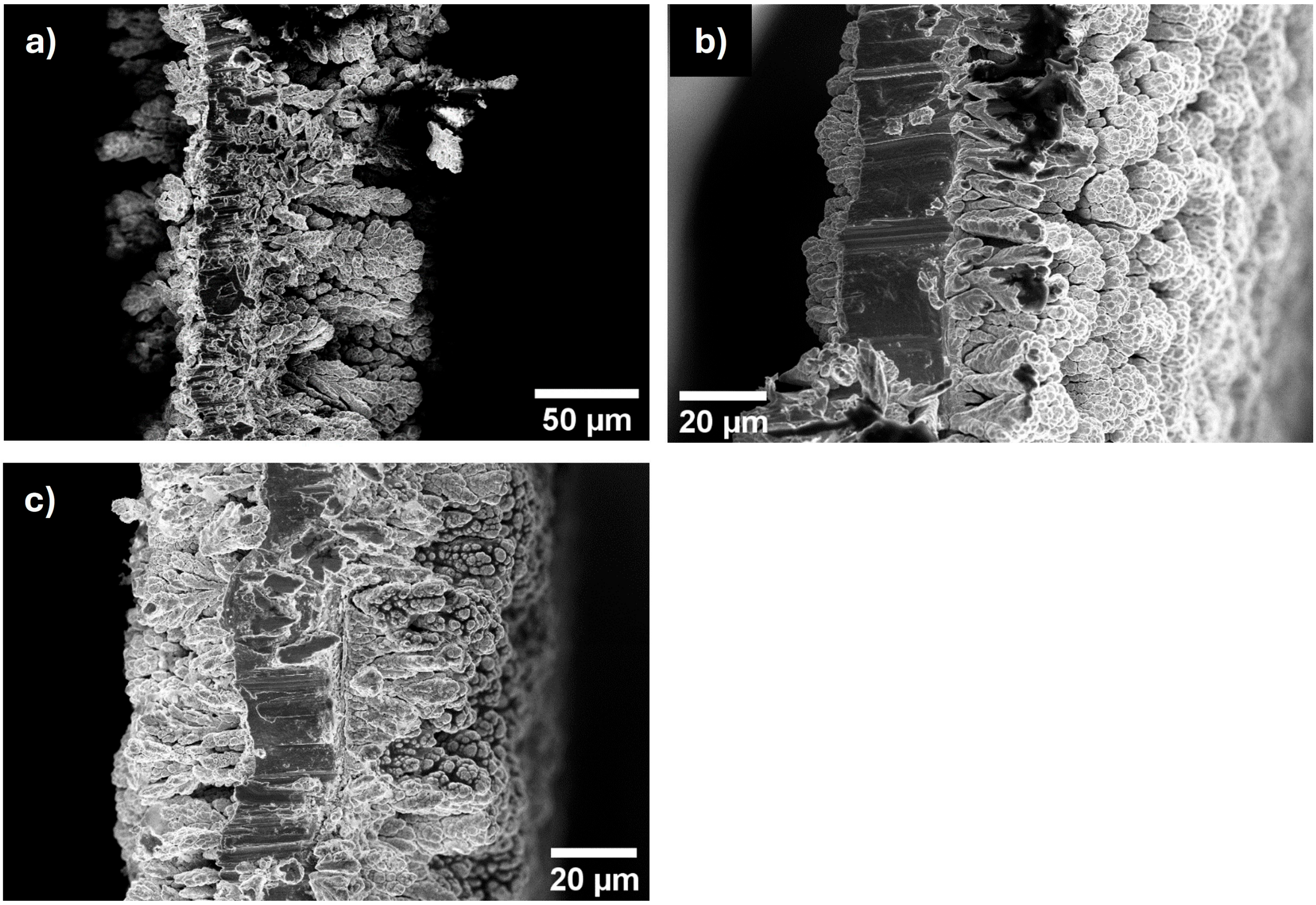

Figure S2. Cross-sectional view SEM images of the Ni-Cu scaffolds fabricated at different electrodeposition currents: a) I = 1 A (0.25 A/cm^2), b) I = 1.6 A (0.40 A/cm^2), c) I = 2.4 A (0.60 A/cm^2). While the thickness of the electrodeposited Ni layer is uniform on the front side, the backside exhibits a strong thickness gradient, ranging from approximately 40 μm near the edges of the Cu foil to only a few nanometers in the central region.

Porosity evaluation

The SEM images were analyzed using a Fiji script [43] to identify micrometric pores generated during electrodeposition. The automated image analysis routine is illustrated in **Fig.S3**: each image is binarized and processed to isolate the microscale pores (which persist after pre-annealing) from the background; a particle identification routine then detects them and extracts their equivalent radius, $\boldsymbol{R = \sqrt{A/\pi}}$,

with an uncertainty u_R = 1px. For each sample, 9 non-overlapping images are acquired in a 3x3 grid, with a magnification (1.471-2.793 μm/px) chosen to include thousands of distinct pores per image.

The combined pore size distributions are shown in **Fig.3b** for the I = 2A samples and in **FigS4a** for the I = 2.4A sample. The latter presents a simple polydispersity, unlike the bimodal distribution of the formers, and it is shifted toward larger pore sizes. To assess local homogeneity, **Fig. S4b-c** shows per-image map of the Sauter radius, $R_{32} = \langle R^3\rangle/\langle R^2\rangle$, corresponding to the radius of a pore with the average surface-to-volume ratio as the distribution. The I=2A samples are homogeneous across different areas and between batches, with sub-micron variability randomly distributed. The I = 2.4A sample presents a similar variability in R_{32}, but with a clear spatial dependence, with a maximum closer to the electrical contact.

https://doi.org/10.3390/nano16150950

For a monolayer of pores like these samples, image analysis can produce a lower bound (excluding the contribution of inter-dendrites voids) for the porosity ε, defined as the ratio between the void volume and the total volume of the object $\varepsilon = V_{void}/V_{tot}$, from the ratio of the areas expressed in pixels: $\varepsilon \geq A_{pores}/A_{image}$. The lower bound is $\varepsilon \geq 70\%$ consistently among the samples, determined using ~~9~~ nine images each to boost the statistics.

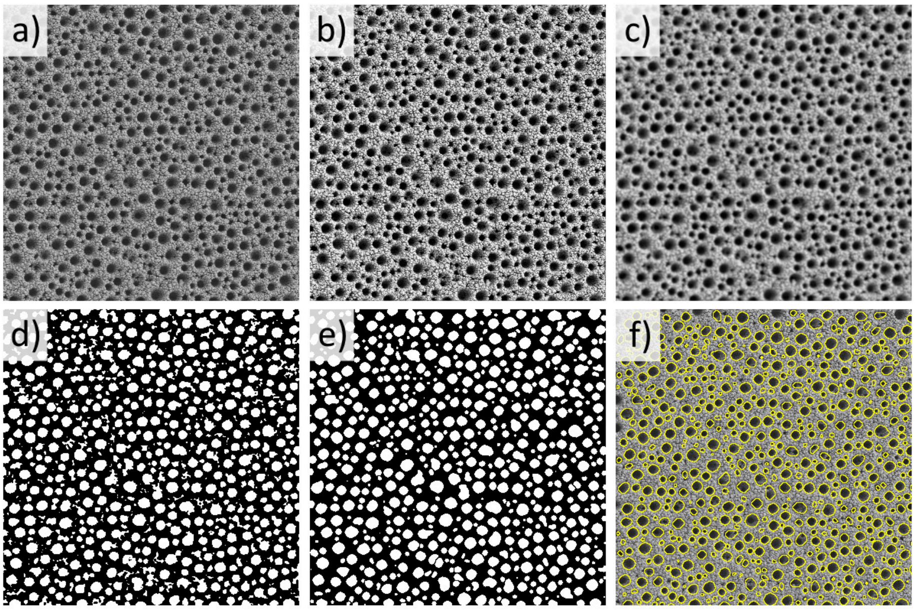

Figure S3. Image analysis routine: (a) portion of a SEM raw image, (b) image whose contrast and brightness have been improved using the local contrast enhancement (CLAHE) algorithm, (c) image cleaned from nanoscale pores with a gaussian blur, (d) binarized image using a local threshold and Otsu algorithm, (e) image treated with a cycle of morphological transformations (closure/erosion/dilation) alternated with the watershed algorithm to separate close pores and smooth their profiles (f) original image overlapped with the contours identified on the pores.

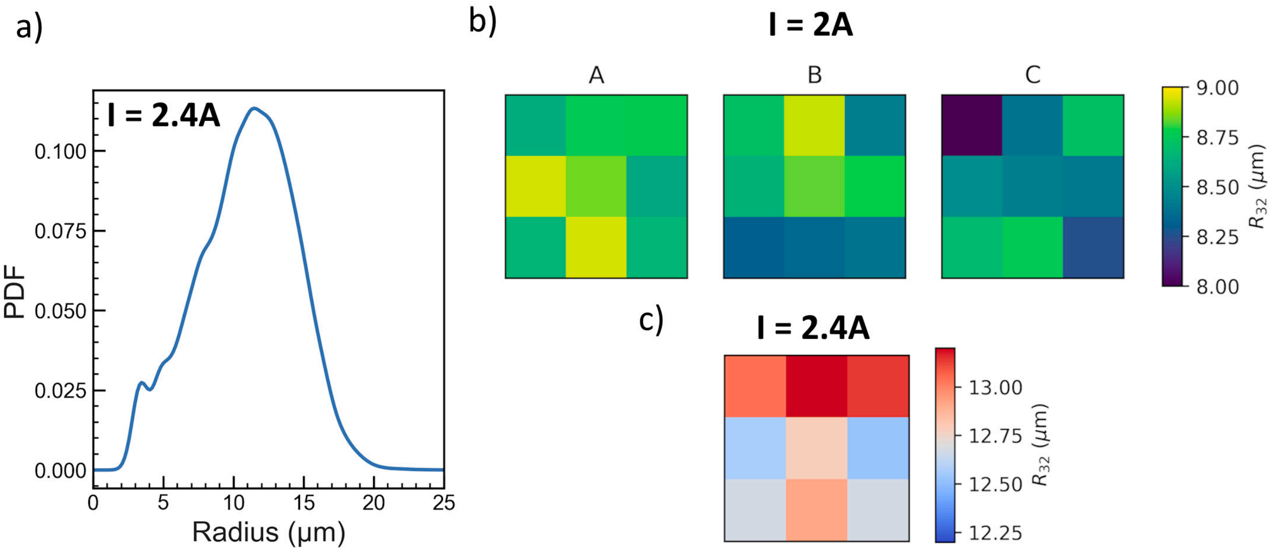

Figure S4. Analysis of pore size distribution: a) combined pore size distribution for the I = 2.4 A sample, presenting a polydisperse distribution of sizes. On the right are shown the maps of the Sauter radius R32 across multiple portions of the samples: b) the I = 2A batches A, B, C; c) the I = 2.4 A sample.

https://doi.org/10.3390/nano16150950

Alloying vs pre-annealing time

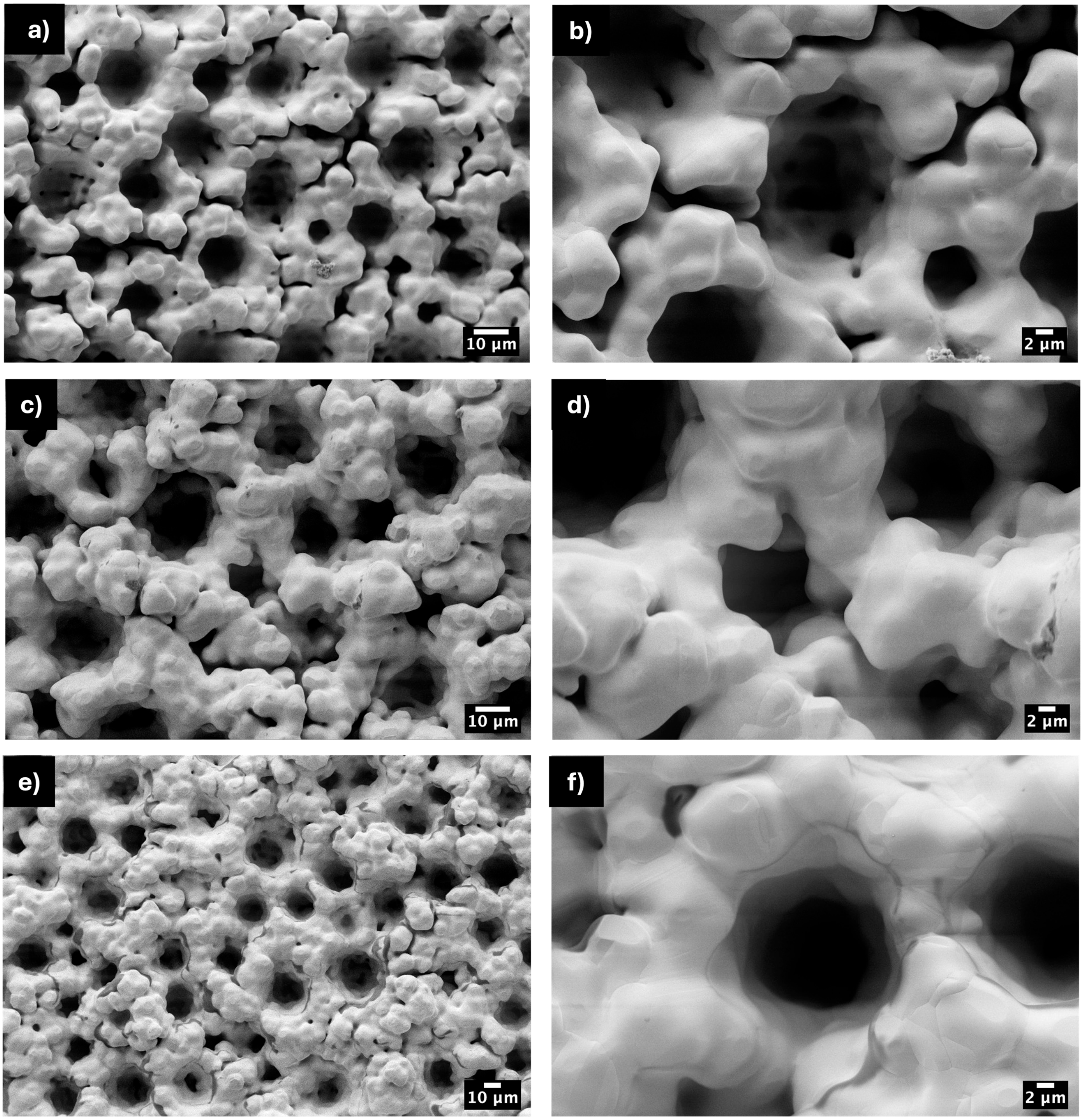

Figure S5. SEM images of the Ni-Cu scaffolds, fabricated with I = 2 A, after different pre-annealing times: a,b) 1 hour (**A0**); c,d) 3 hours (**A3**); e,f) 7 hours (**A7**).

https://doi.org/10.3390/nano16150950

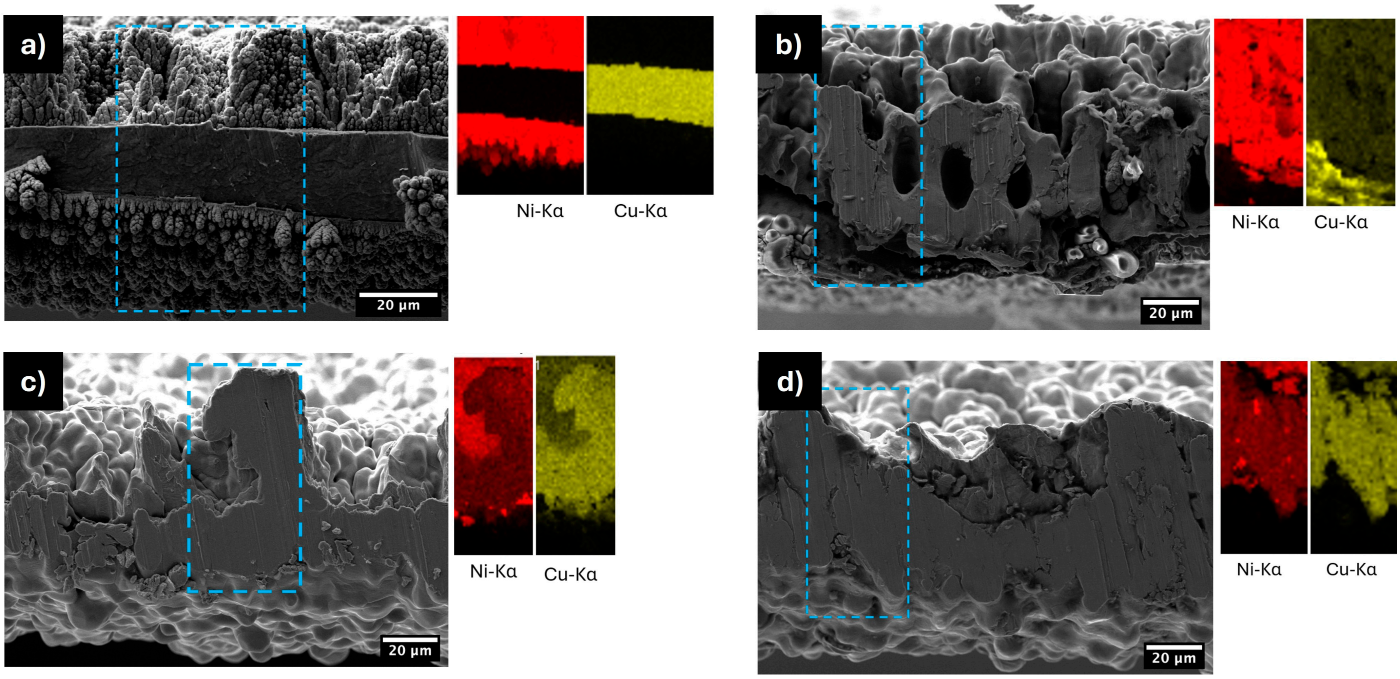

Figure S6. EDS Maps of the Ni-Cu scaffolds fabricated with *I* = 2 A after different pre-annealing times: a) no pre-annealing (**A0**)**;** b) 1 hour (**A1**); c) 3 hours (**A3**); and d) 7 hours (**A7**).

Supplementary XRD analysis of Ni-Cu alloy formation

Wide-range specular XRD scans, reported in **Figure S7**, were used to verify the diffraction features discussed in the main text. The analysis is therefore based on peak positions, peak shape, and their relative evolution with pre-annealing time.

The wide scans show the fcc reflection sequence of the Ni-Cu scaffold. The asymmetric shape of the metal reflections motivated the HRXRD analysis reported in **Figure 6**. In the HRXRD fits, multiple components were introduced only when required to reproduce the asymmetric profiles. At 3 h, the Ni(111) and Ni(200) reflections show a different component distribution, despite being measured in the same specular geometry and at comparable Bragg angles. This supports the assignment of the difference to non-uniform lattice expansion and/or residual strain rather than to a substantially different probed volume.

For the composition estimate, the fitted peak positions were converted into fcc lattice parameters and then into Cu atomic fraction using Vegard-type interpolation between pure Ni and pure Cu. These values should be regarded as semi-quantitative, because residual strain, defects, and overlapping alloyed domains can also shift the fitted peak positions. The Cu-content scale is therefore used to compare relative trends between samples, rather than as an absolute chemical analysis.

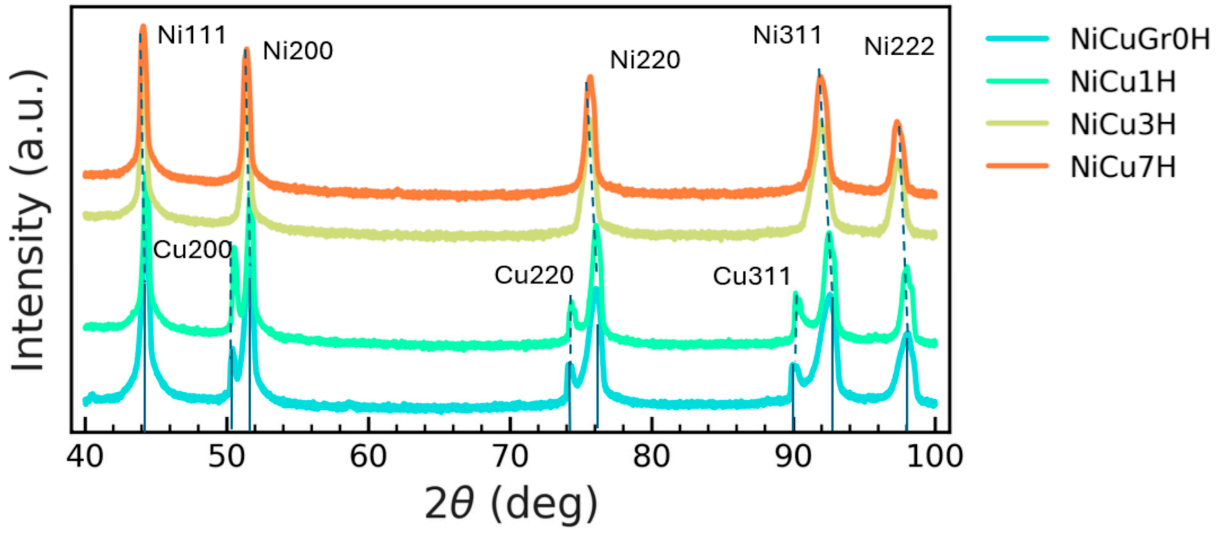

Figure S7. Specular XRD patterns of Ni-Cu scaffolds as a function of pre-annealing time. Patterns are vertically offset for clarity. The main FCC Ni and Cu reflections are labeled.

Specular/GI-XRD analysis of the Ni/Cu(311) reflection

The Ni/Cu(311) reflection was selected for the geometry-dependent analysis because it is the most intense feature in the specular/GI-XRD measurements and is located at relatively high diffraction angle, where overlapping contributions are more separated. The profiles were compared in specular geometry and at GI angles of 10° and 4°. Because the scaffolds are rough, porous, and three-dimensional, these measurements were not converted into absolute probing depths.

The specular/GI comparison was used only to evaluate whether the distribution of lattice spacings depends on measurement geometry. At shorter pre-annealing times, the broader specular profile indicates that the full illuminated volume contains a wider distribution of Ni-Cu lattice spacings than the regions emphasized in GI geometry. With increasing pre-annealing time, the profiles measured in the different geometries become closer in peak position and width. This indicates that the compositional distribution becomes less dependent on vertical position within the scaffold, although HRXRD still resolves local heterogeneity.

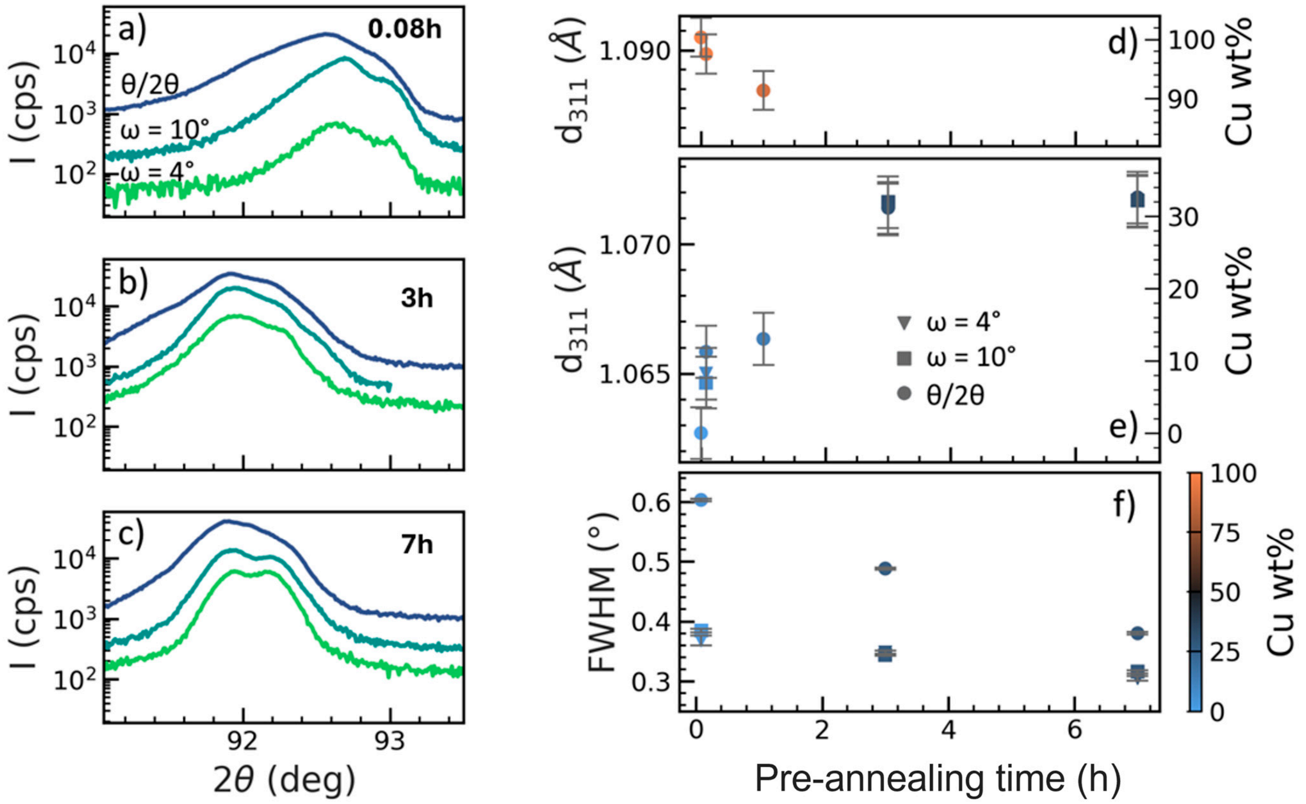

Figure S8. (a-c) Comparison of the Ni-Cu-(311) reflection profiles collected in specular geometry and at GI angles of 10° and 4°-*vs* pre-annealing times. (d, e) Interplanar spacing and FWHM values extracted from the 311 reflection as a function of pre-annealing time. The corresponding estimated Cu content is reported on the secondary axis or color scale (f).

Graphene on Ni-Cu scaffold

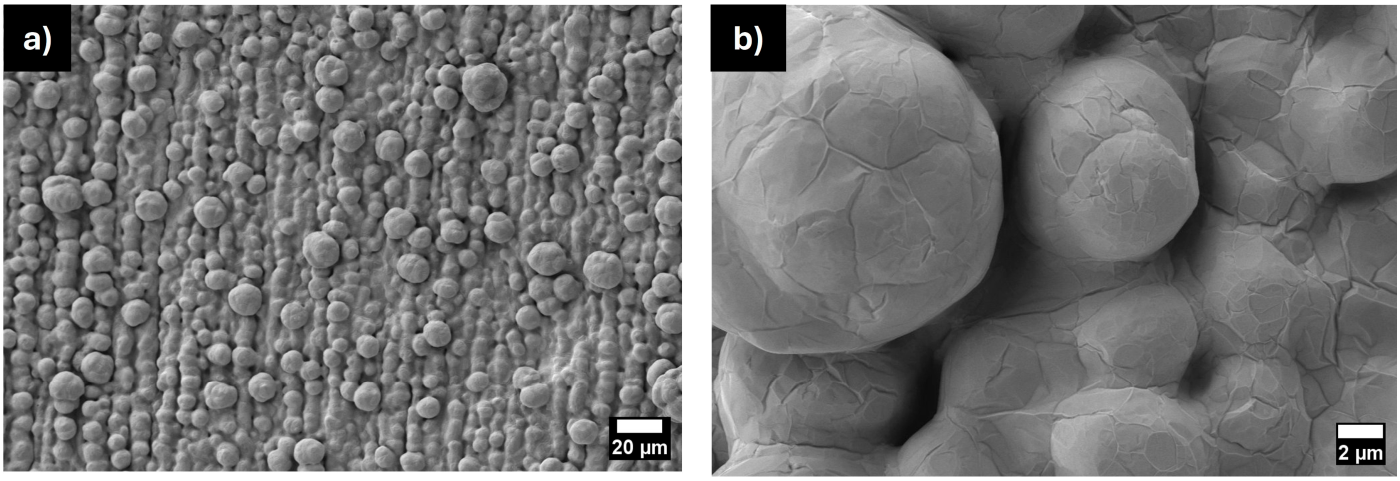

Figure S9. SEM images of the backside of **A0Gr** sample.

Table S1. Weighted average of the spectral parameter with their weighted standard deviations for **A1Gr-A7Gr**, estimated from five random areas each.

	A1Gr	A3Gr	A7Gr
ω_D (cm^{-1})	1343.29+/-4.05	1354.53+/-1.35	1354.75+/-2.91
ω_G (cm^{-1})	1581.86+/-0.57	1577.93+/-0.38	1578.02+/-0.62
ω_{2D} (cm^{-1})	2709.09+/-0.81	2705.27+/-0.41	2705.68+/-0.97
I_D/I_G	0.03+/-0.00	0.03+/-0.01	0.01+/-0.00
I_{2D}/I_G	0.58+/-0.05	0.41+/-0.03	0.40+/-0.02
A_D/A_G	0.07+/-0.07	0.05+/-0.01	0.02+/-0.00
A_{2D}/A_G	1.30+/-0.18	0.97+/-0.07	1.03+/-0.05
$FWHM_G$ (cm^{-1})	24.74+/-1.18	24.05+/-1.04	21.87+/-0.57
$FWHM_{2D}$ (cm^{-1})	78.77+/-5.42	78.87+/-1.62	75.90+/-1.28

https://doi.org/10.3390/nano16150950

3D Etched Graphene

https://doi.org/10.3390/nano16150950

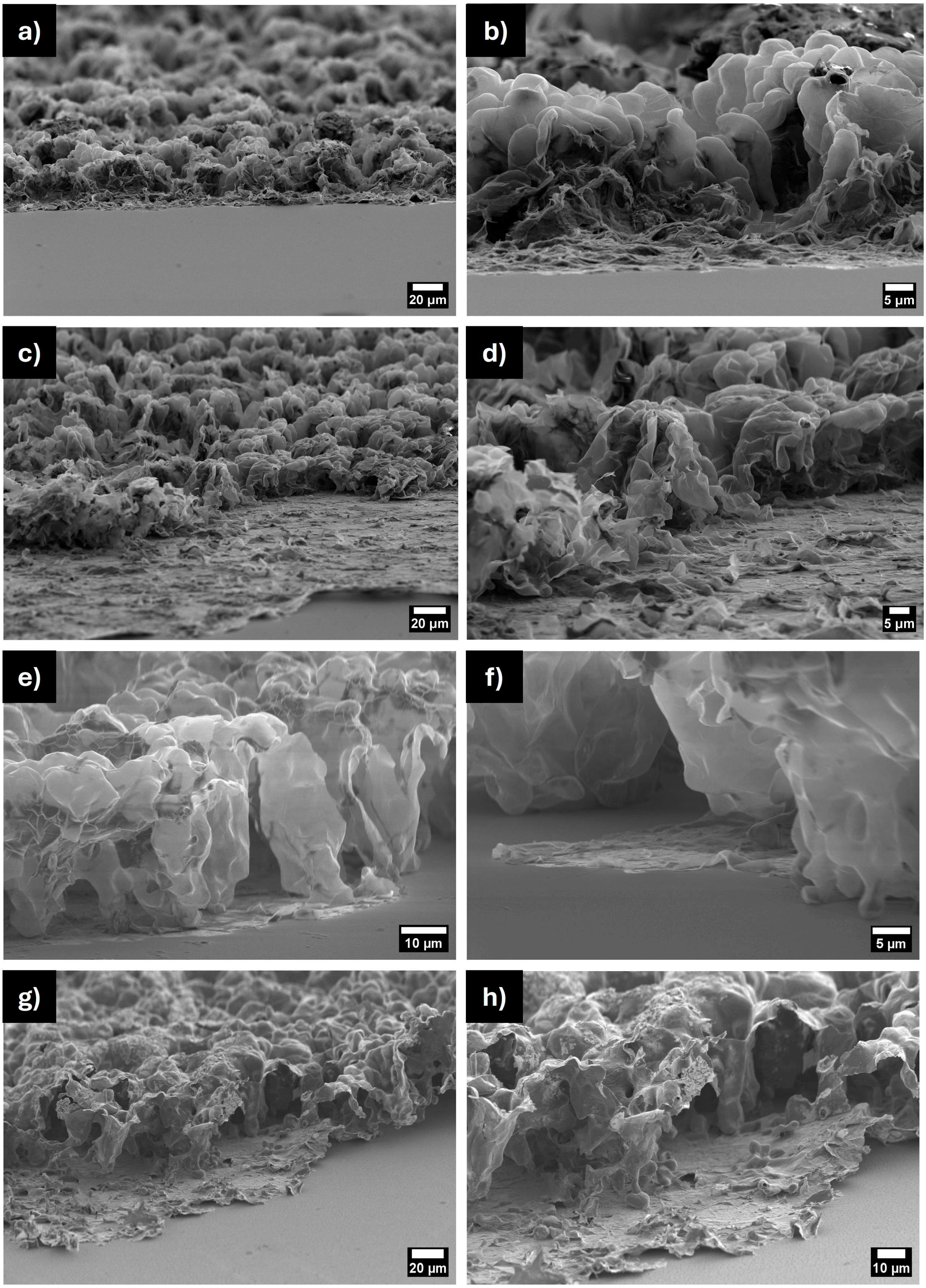

Figure S10..

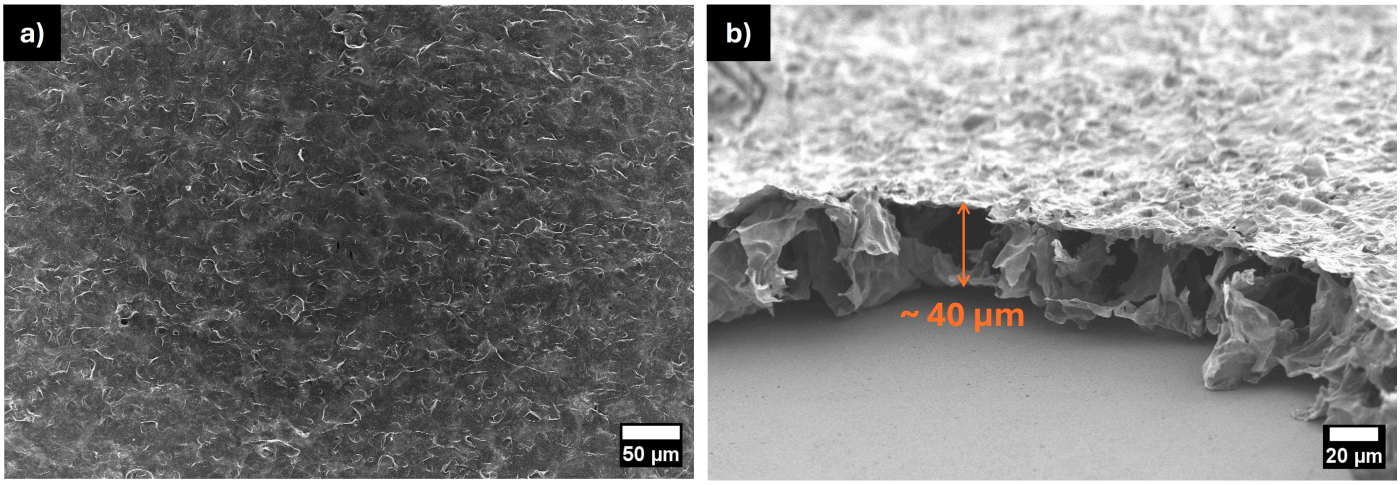

Figure S11. a) top and b) cross-sectional view SEM images freestanding graphene foam **Gr7** from its backside.

Multi-Peak fitting procedure for Raman spectra

The baseline-corrected Raman spectra were fitted using a multi-component model comprising pseudo-Voigt functions for the G (~1580 cm^{-1}) and D (~1350 cm^{-1}) bands, four Lorentzian functions for the 2D band (~2750 cm^{-1}), and Gaussian functions for the defect-related D′ (~1620 cm^{-1}), D″ (~1500 cm^{-1}), and F (~1450 cm^{-1}) bands (**Figure S12**).

The G band, centered around 1580 cm−1, is associated with the in-plane stretching mode of sp^2-bonded carbon and is modeled using a Voigt function to account for both homogeneous and inhomogeneous broadening. The D band, associated with intervalley defect-induced scattering, was fitted using the same line shape. Its intensity reflects the presence of structural disorder in the sp^2 carbon network, including point defects, grain boundaries, edges, and local perturbations of bond polarizability.

Moreover, Turner et al. [44], demonstrated that pseudo-Voigt functions provide more reliable fits than Lorentzian functions, and pseudo-Voigt fits are also more consistent across different software packages when using the same fitting methodology. A secondary defect-related contribution, the D′ band at approximately 1620 cm−1, is described by a Gaussian function and is introduced to account for disorder-activated scattering when the G-band wavenumber is constrained during fitting. In highly defective/disordered/amorphous carbon materials, this contribution may partially overlap with the G band, making the deconvolution of the two features less straightforward. Additional disorder-related contributions, namely the D″ and F bands, were also included as Gaussian components when required by the experimental spectra.

The 2D band of graphene is a second-order double-resonance process, and its shape depends strongly on the electronic band structure. Since four symmetry-allowed double-resonance scattering pathways contribute to this feature, the resulting lineshape can be described as a superposition of four closely spaced Raman sub-peaks associated with distinct electronic transitions. Accordingly, the 2D band was modeled using four Lorentzian components, an approach commonly adopted for few-layer graphene [45,37].

Following an initial fitting cycle, the amplitudes of the D′, D″, and F bands were compared against a predefined detection threshold. Components with amplitudes below this threshold were considered statistically insignificant and removed from the model, after which the spectrum was refitted. For each retained peak, the position, height, integrated area, and full width at half maximum (FWHM) were extracted. For the 2D band, the reported FWHM

https://doi.org/10.3390/nano16150950

corresponds to that of the overall four-component envelope. Uncertainties arising from baseline estimation and subtraction were propagated to all fitted parameters. The position of the 2D band ($\boldsymbol{\omega_{2D}}$) was calculated as the area-weighted average of the four Lorentzian sub-peaks:

$$\boldsymbol{\omega_{2D}} = \sum \boldsymbol{position_{peak\_i}} \times \boldsymbol{A_{peak\_i}} \,/\, \sum \boldsymbol{A_{peak\_i}}.$$

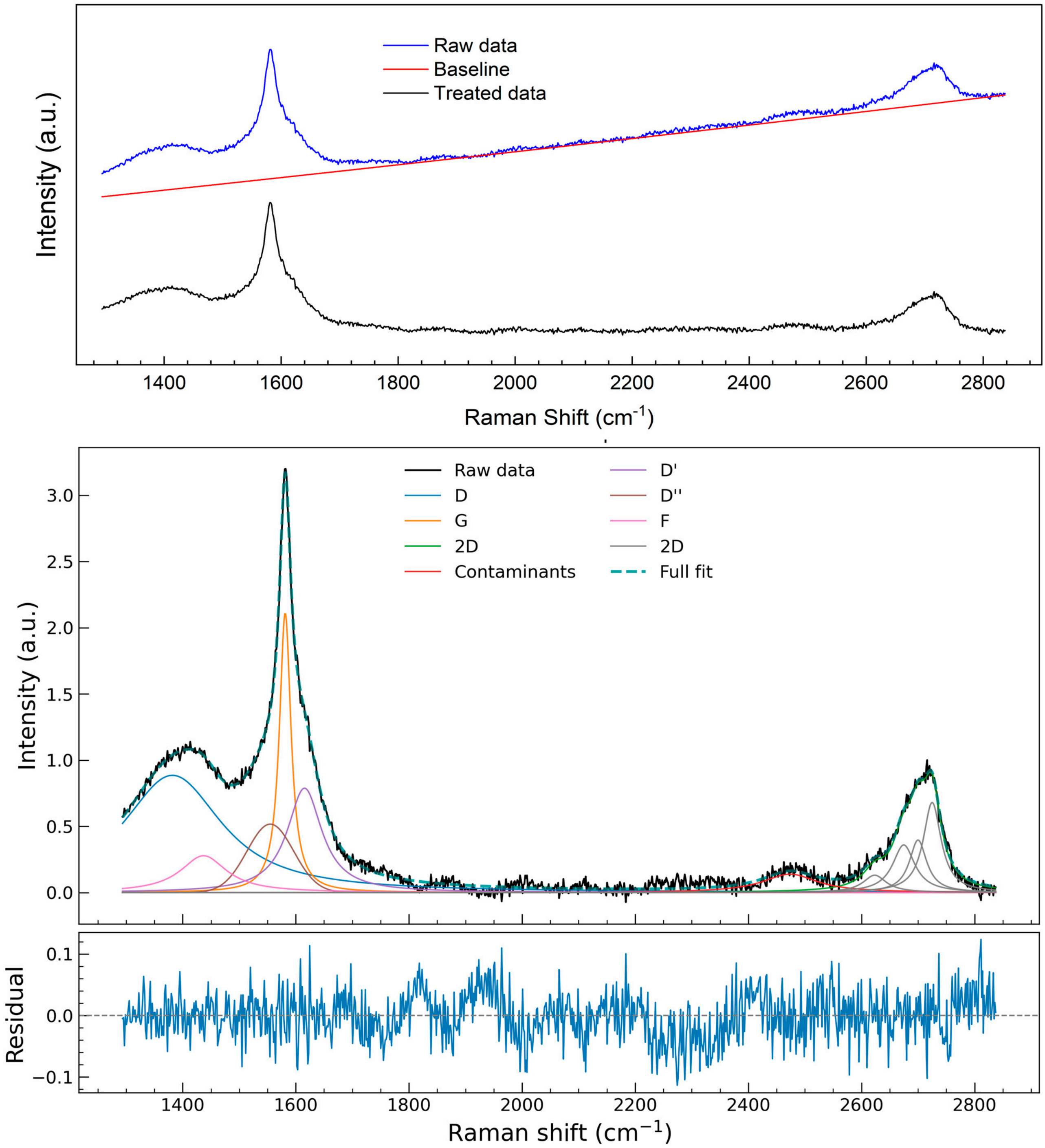

Figure S12. Example of Raman spectra for **Gr0**. a) Photoluminescence baseline fitting and subtraction from raw data. The baseline has been fitted to the data in the ranges [1800, 2400] - [2800, 2850] cm^{-1}, and interpolated/extrapolated to the regions containing peaks. b) Multiple peak fitting on the treated data, in units of I_{2D}. The individual components are highlighted by colored lines, and the full fit is represented by a dashed line. c) Residuals between the data and the fit.

Raman results

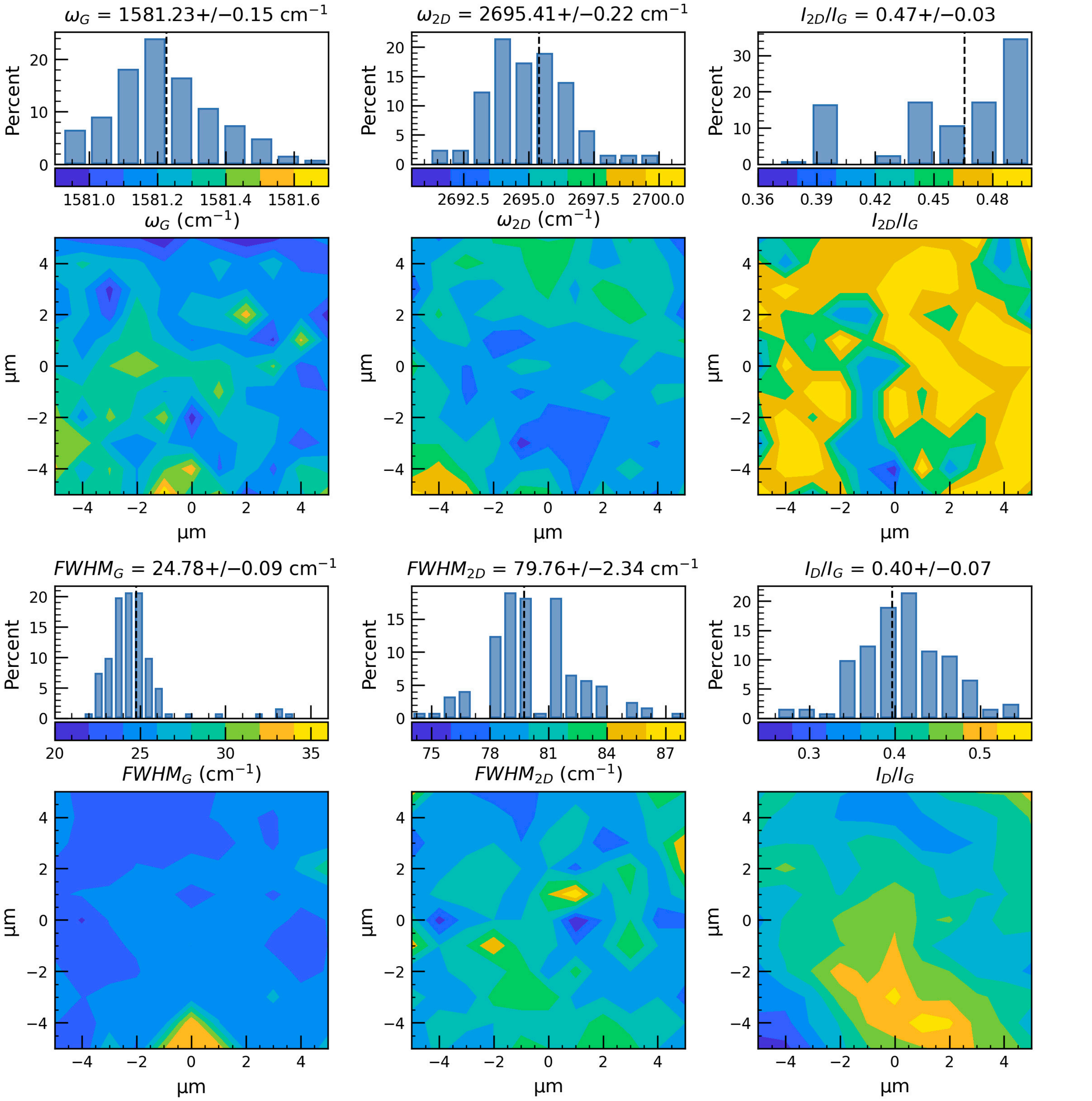

Figure S13. 10 × 10 µm² Raman maps and corresponding statistical distributions of the G and 2D band parameters across the scanned area of sample **Gr0**. The parameters were obtained from baseline-corrected Raman spectra and extracted through peak fitting. Dashed lines in the histograms denote the weighted mean values, which are reported above each plot together with their corresponding standard deviations.

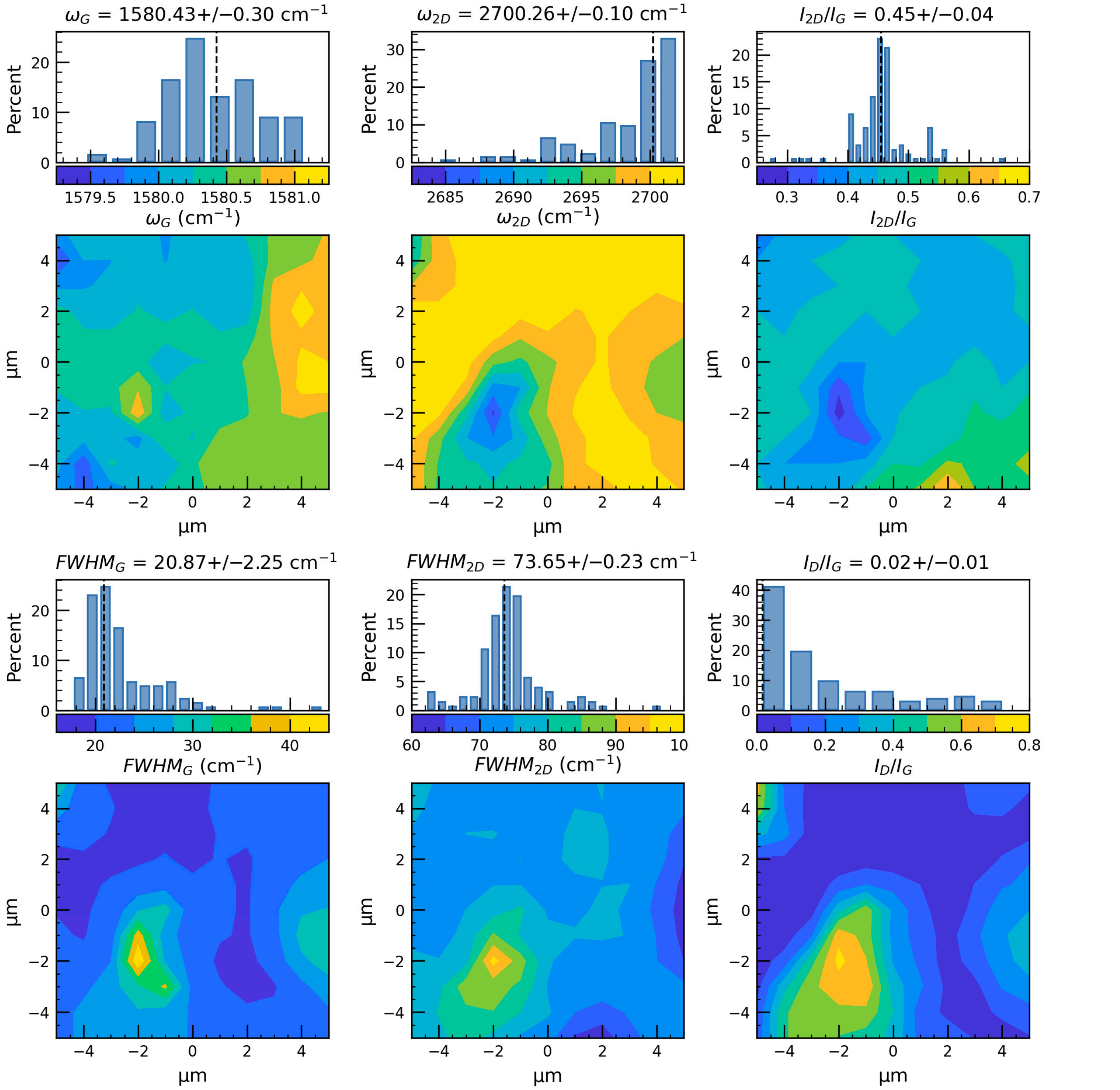

Figure S14. 10 × 10 μm² Raman maps and corresponding statistical distributions of the G and 2D band parameters across the scanned area of sample **Gr1**. The parameters were obtained from baseline-corrected Raman spectra and extracted through peak fitting. Dashed lines in the histograms denote the weighted mean values, which are reported above each plot together with their corresponding standard deviations.

https://doi.org/10.3390/nano16150950

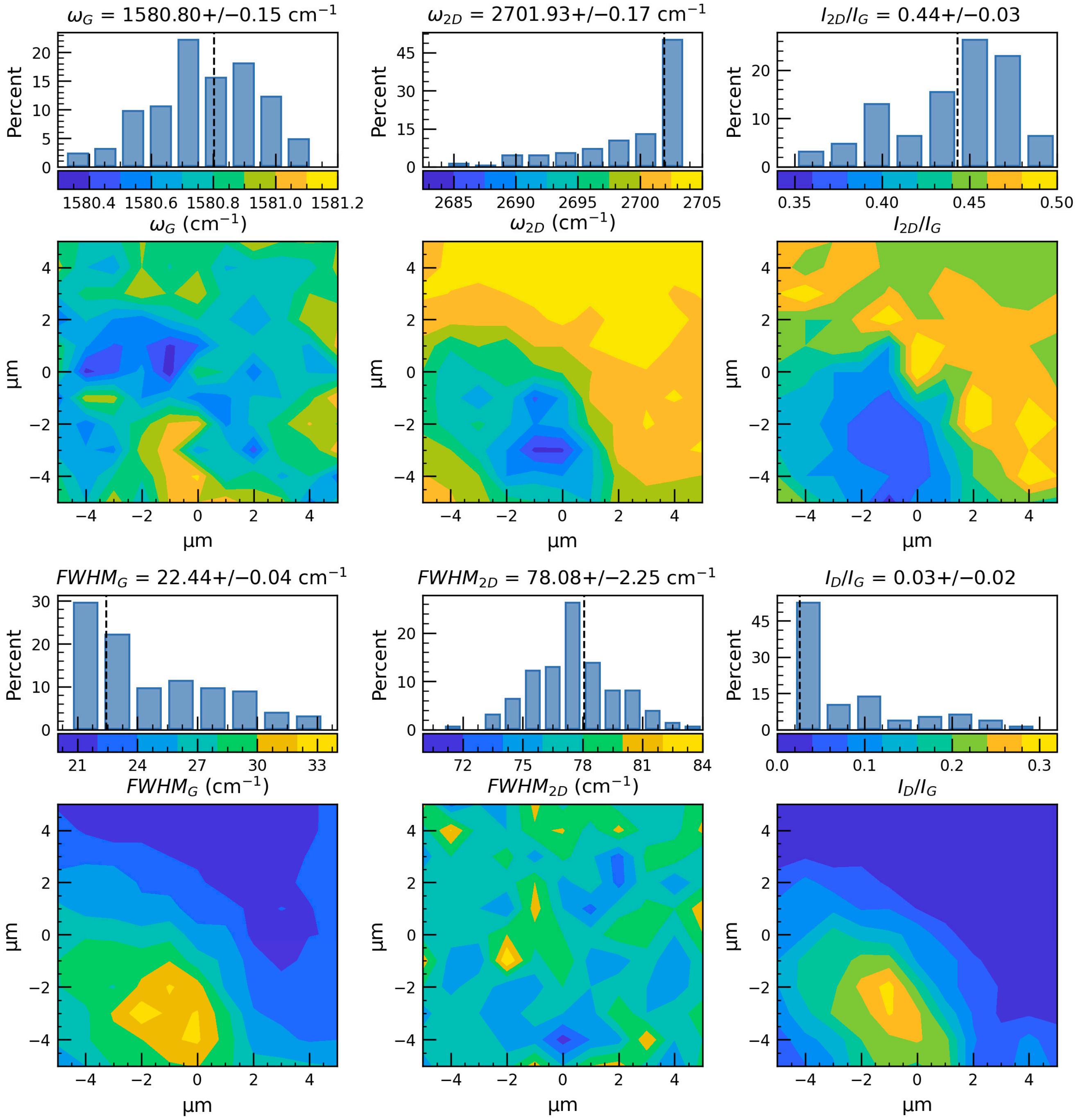

Figure S15. 10 × 10 µm² Raman maps and corresponding statistical distributions of the G and 2D band parameters across the scanned area of sample **Gr3**. The parameters were obtained from baseline-corrected Raman spectra and extracted through peak fitting. Dashed lines in the histograms denote the weighted mean values, which are reported above each plot together with their corresponding standard deviations.

https://doi.org/10.3390/nano16150950

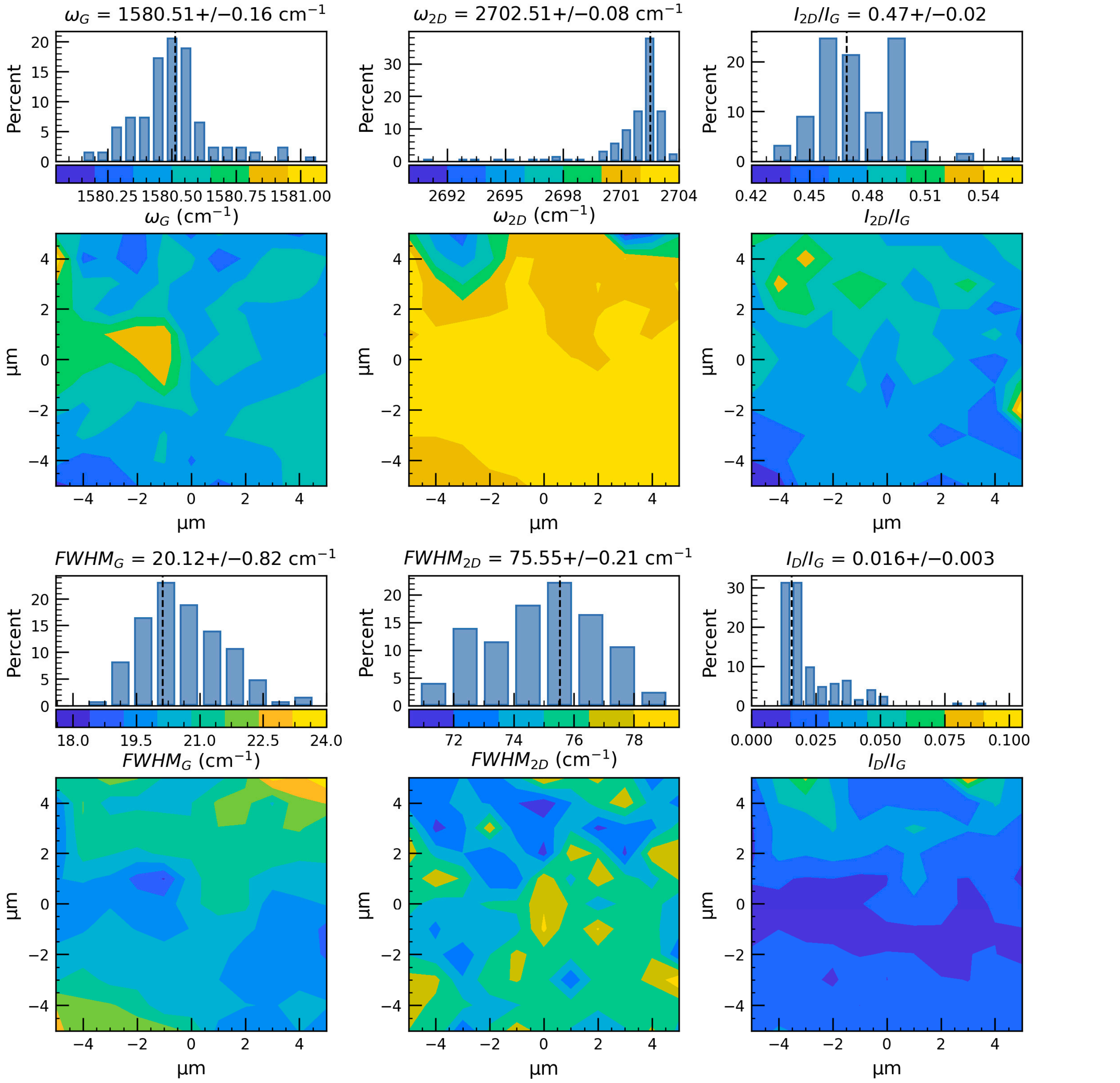

Figure S16. 10 × 10 μm² Raman maps and corresponding statistical distributions of the G and 2D band parameters across the scanned area of sample **Gr7**. The parameters were obtained from baseline-corrected Raman spectra and extracted through peak fitting. Dashed lines in the histograms denote the weighted mean values, which are reported above each plot together with their corresponding standard deviations.

https://doi.org/10.3390/nano16150950

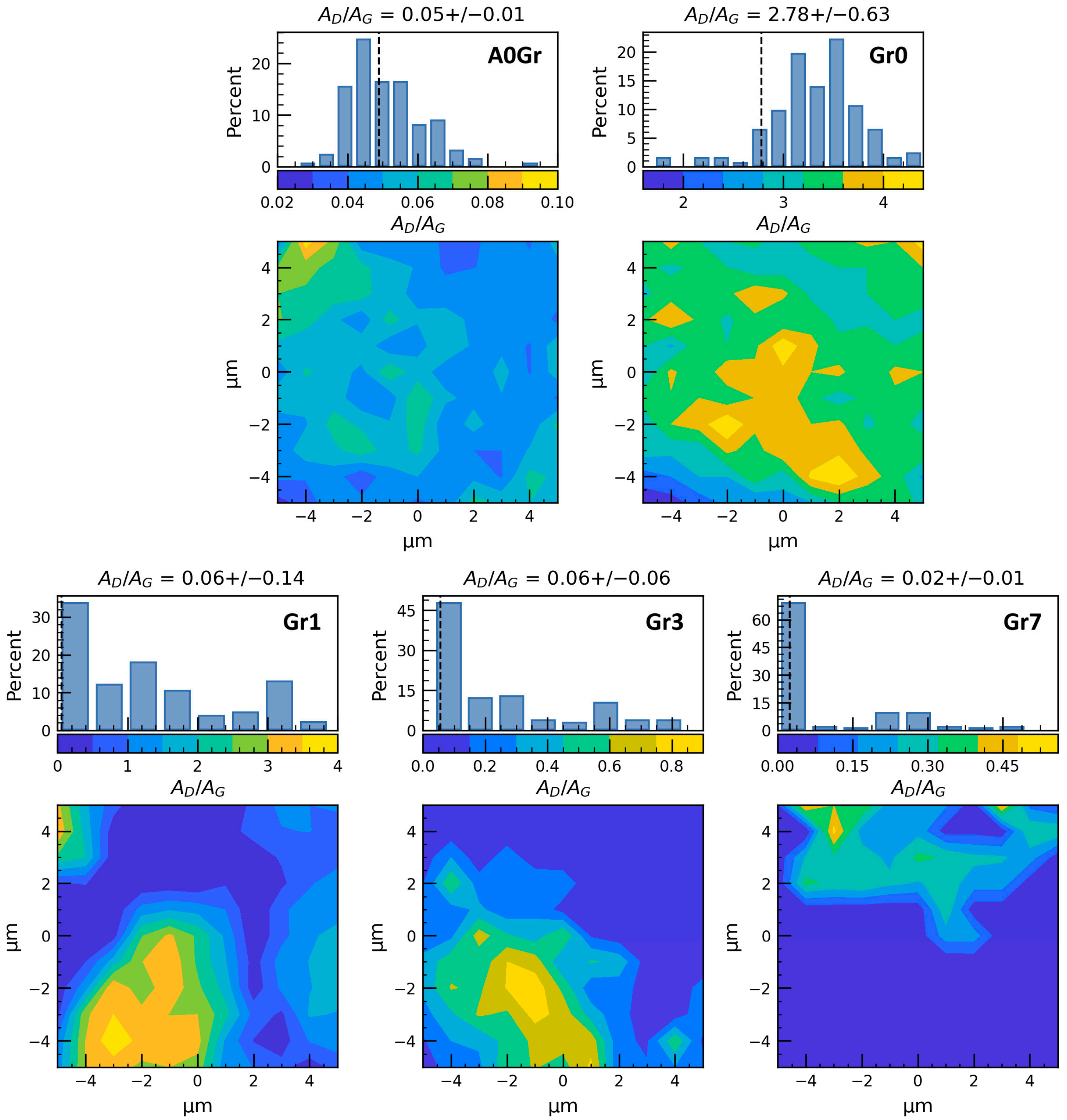

Figure S17. 10 × 10 µm² Raman maps and corresponding statistical distributions of the A_D/A_G ratio across the scanned area of the samples (from top left to bottom right): **A0Gr**, **Gr0**, **Gr1**, **Gr3**, **Gr7**. The parameters were obtained from baseline-corrected Raman spectra and extracted through peak fitting. Dashed lines in the histograms denote the weighted mean values, which are reported above each plot together with their corresponding standard deviations.

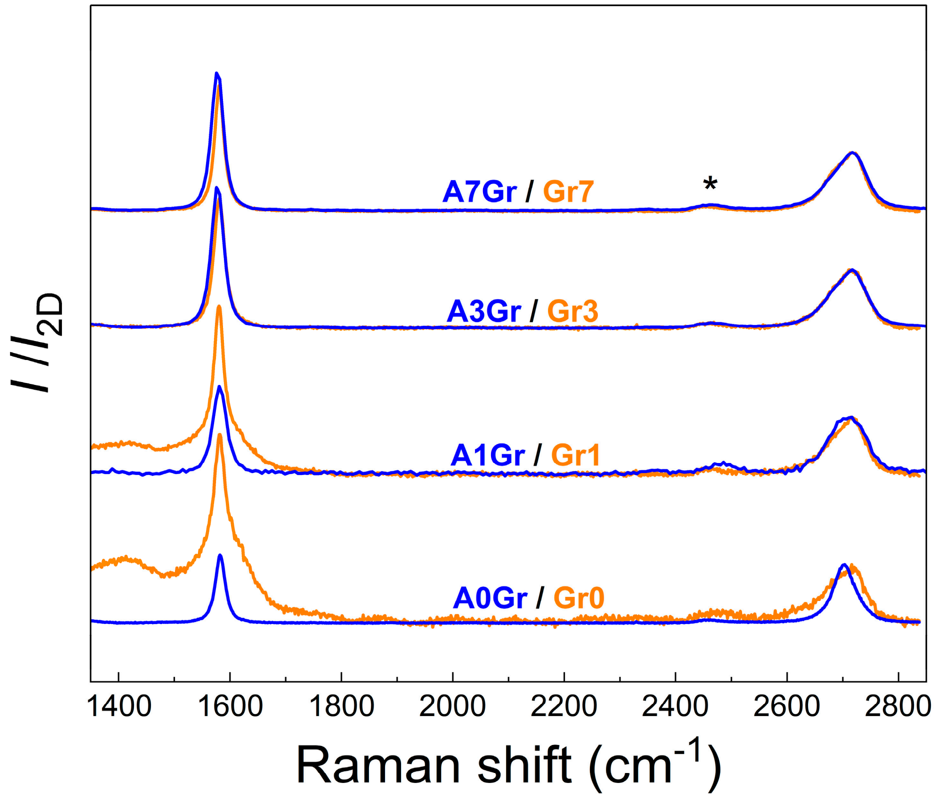

Figure S18. Representative Raman spectra for each sample before and after wet etching of the Ni-Cu scaffold. The spectra from maps have been selected to exhibit with I_{2D}/I_G values comparable to the weighted average values reported in Tables 1 and S1. Data are normalized to 2D peak intensity and arbitrarily shifted for each sample to allow a clearer comparison. The main peak components are labelled; the asterisk marks a peak attributed to contaminants on the sample.

Optical Measurements

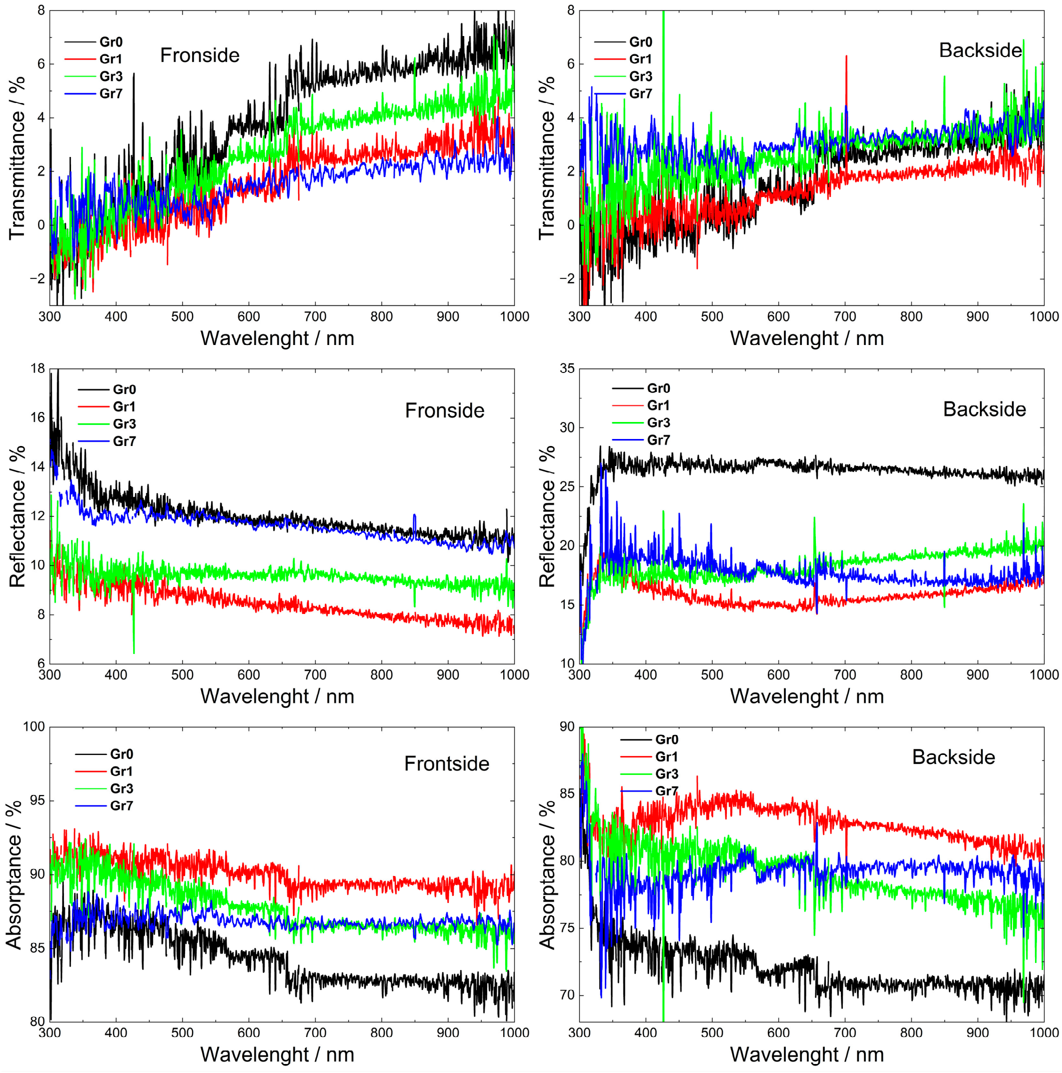

Figure S19. From top to bottom, UV–Vis–NIR Transmittance, Reflectance and Absorptance spectra measured from the front (left) and back (right) sides for the 3D graphene foam. The high noise level is due to the use of the integrating sphere, which decreases the intensity of the light reaching the detector. Consequently, the signal-to-noise ratio is reduced, making the measurements more sensitive to noise.

https://doi.org/10.3390/nano16150950
